# Thr 163 Phosphorylation Causes Mcl-1 Stabilization when Degradation Is Independent of the Adjacent GSK3-Targeted Phosphodegron, Promoting Drug Resistance in Cancer

**DOI:** 10.1371/journal.pone.0047060

**Published:** 2012-10-09

**Authors:** Shanna K. Nifoussi, Julie A. Vrana, Aaron M. Domina, Alfredo De Biasio, Jingang Gui, Mark A. Gregory, Stephen R. Hann, Ruth W. Craig

**Affiliations:** 1 Department of Pharmacology and Toxicology, Geisel School of Medicine at Dartmouth, Hanover, New Hampshire, United States of America; 2 Norris Cotton Cancer Center, Dartmouth-Hitchcock Medical Center, Lebanon, New Hampshire, United States of America; 3 Department of Cell and Developmental Biology, Vanderbilt University School of Medicine, Nashville, Tennessee, United States of America; University of Nebraska Medical Center, United States of America

## Abstract

The antiapoptotic Bcl-2 family member Mcl-1 is a PEST protein (containing sequences enriched in proline, glutamic acid, serine, and threonine) and is subject to rapid degradation via multiple pathways. Impaired degradation leading to the maintenance of Mcl-1 expression is an important determinant of drug resistance in cancer. Phosphorylation at Thr 163 in the PEST region, stimulated by 12-O-tetradecanoylphorbol acetic acid (TPA)-induced activation of extracellular signal-regulated kinase (ERK), is associated with Mcl-1 stabilization in BL41-3 Burkitt lymphoma cells. This contrasts with the observation that Thr 163 phosphorylation in normal fibroblasts primes glycogen synthase kinase (GSK3)-induced phosphorylation at Ser 159, producing a phosphodegron that targets Mcl-1 for degradation. In the present follow-up studies in BL41-3 cells, Mcl-1 degradation was found to be independent of the GSK3-mediated pathway, providing a parallel to emerging findings showing that Mcl-1 degradation through this pathway is lost in many different types of cancer. Findings in Mcl-1-transfected CHO cells corroborated those in BL41-3 cells in that the GSK3-targeted phosphodegron did not play a major role in Mcl-1 degradation, and a phosphomimetic T163E mutation resulted in marked Mcl-1 stabilization. TPA-treated BL41-3 cells, in addition to exhibiting Thr 163 phosphorylation and Mcl-1 stabilization, exhibited an ∼10-fold increase in resistance to multiple chemotherapeutic agents, including Ara-C, etoposide, vinblastine, or cisplatin. In these cancer cells in which Mcl-1 degradation is not dependent on the GSK3/phosphodegron-targeted pathway, ERK activation and Thr 163 phosphorylation are associated with pronounced Mcl-1 stabilization and drug resistance – effects that can be suppressed by inhibition of ERK activation.

## Introduction

Increased expression of Mcl-1, stimulated by growth factors and other environmental signals, promotes viability and allows the amplification and function of cell types and lineages needed by the organism [1]–[4]. Mcl-1 downregulation, in turn, curbs these processes and induces death in damaged, nonfunctional, or senescent cells [1], [5]–[7]. Maintenance of elevated Mcl-1 expression is associated with drug resistance and poor prognosis in a variety of cancers [8]–[15]. Agents that induce Mcl-1 turnover, and inhibitors designed to target the protein, can promote tumor cell death [16]–[20].

**Figure 1:**
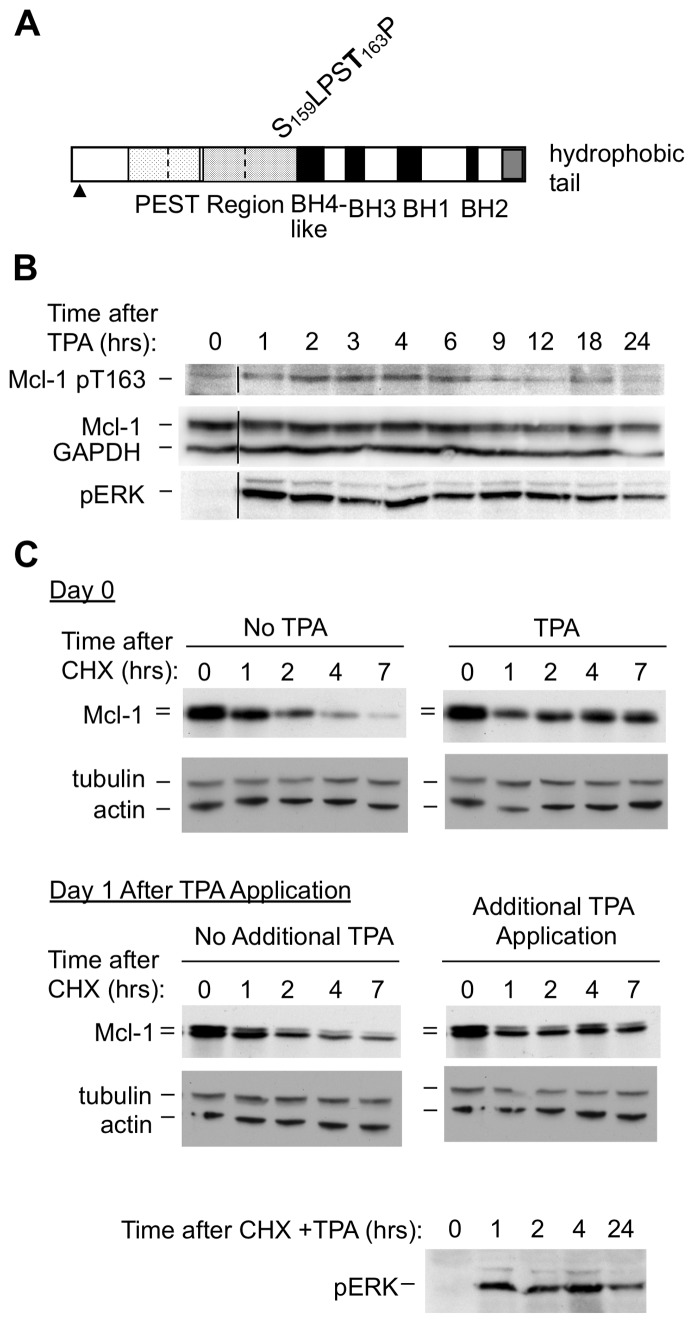
TPA-induced ERK activation, Thr 163 phosphorylation, and Mcl-1 stabilization occur rapidly and are subsequently downregulated. A: The phosphorylation sites at Thr 163 and Ser 159 in the human Mcl-1 protein are diagrammed. Thr 163 is subject to phosphorylation by MAP kinases, as seen upon TPA-induced ERK activation in BL41-3 cells. Ser 159 is subject to phosphorylation by GSK3. Mcl-1 is a PEST protein subject to rapid turnover, the half-life of decay being ∼3 hours in BL41-3 cells (stippling of lesser versus greater density indicates poor PEST and potential PEST sequences; PESTFIND). However, Mcl-1 exhibits striking stabilization upon TPA-induced ERK activation and Thr 163 phosphorylation in these cells. Mcl-1 is also subject to another posttranslational modification involving truncation of the extreme N-terminus ([28] indicated with an arrowhead). This results in a closely spaced 42/40 kd doublet, where the lower 40 kd band lacks ∼16 amino acid residues and is the most abundant band present in BL41-3 cells. The doublet is best visualized on large format electrophoresis gels (Fig. S1B) but can sometimes be detected on standard gels (Fig. 1C). As Thr 163 phosphorylation does not prevent N-terminal truncation, Mcl-1 stabilization in the presence of this phosphorylation is seen as slowed decay of the 40 kd band. **B:** BL41-3 cells were either left untreated or exposed to TPA (5 nM) for the indicated times, and monitored for expression of Thr 163 phosphorylated Mcl-1 (Mcl-1 pT163), total Mcl-1, phosphorylated ERK (pERK), and GAPDH (ChemiDoc). All samples were run at the same time and subjected to the same autoradiographic exposure, where the black vertical line in this and subsequent figures indicates that lanes have been rearranged to facilitate comparison. Thr 163 phosphorylation was also induced by lower concentrations of TPA (e.g., 1 nM; Fig. S1C), and was inhibited by U0126 (Fig. S1D). m **C:** BL41-3 cells were either left untreated or exposed to TPA (1 nM). Some cells were immediately exposed to CHX to monitor Mcl-1 protein decay on Day 0. Additional TPA-treated cells were incubated for 24 hours (Day 1 after TPA Addition) and then exposed to CHX, where a portion of these cells was retreated with TPA at this time. The untreated cell sample (Time 0) is identical for each pair of Western blots. The blot at the bottom confirmed that pERK was reduced at 24 hours.

Mcl-1 was identified based on increased transcription in ML-1 human myeloblastic leukemia cells induced to differentiate upon exposure to TPA [21], [22]. Mcl-1 is also regulated post-translationally (Fig. 1A), as observed in the BL41-3 Burkitt lymphoma cell line in which endogenous Mcl-1 is amplified and overexpressed [1], [23], [24]. Mcl-1 is a PEST protein and is normally subject to rapid turnover. However, exposure of BL41-3 cells to TPA results in activation of the mitogen-activated protein (MAP) kinase ERK, along with an ERK-dependent increase in Mcl-1 phosphorylation at Thr 163 and markedly slowed degradation of the Mcl-1 protein [25], [26].

Mcl-1 is also subject to phosphorylation by GSK3 [20], [27]–[34]. In fact, MAP kinase-induced phosphorylation at Thr 163 provides a priming site for GSK3-induced phosphorylation at Ser 159, as has been shown in normal mouse fibroblasts exposed to ultraviolet (UV) irradiation [27]. In this system, phosphorylation at the MAP kinase phosphorylation site (Thr 163) is carried out by c-Jun N-terminal kinase (JNK); ensuing phosphorylation at the GSK3 phosphorylation site (Ser 159) results in the production of a phosphodegron that targets Mcl-1 for degradation by E3 ubiquitin ligases containing F-box proteins [28], [32]–[34]. Mcl-1 can also be degraded by a variety of other pathways, such as via the BH3-containing E3 ubiquitin ligase MULE (also called Arf-BP/Lasu1/Huwe1 [35], [36]), the anaphase promoting complex [37], and ubiquitin-independent mechanisms [38].

Emerging findings indicate that a reduction in Mcl-1 degradation via the above GSK3-induced pathway contributes to drug resistance in cancer. For example, GSK3 inactivation in breast cancer patient samples is associated with abundant Mcl-1 expression and poor outcome [20], [32]. Similarly, reduced Mcl-1 degradation via the GSK3/phosphodegron-targeted pathway has been implicated in chronic lymphocytic leukemia [13], [39], [40]. A variety of drug-resistant cancers exhibit inactivation of the F-box protein FBW7, which lies downstream of phosphorylation events such as those induced by GSK3 [33], [34]. In other cases, cancer cells exhibit increased expression of a deubiquitinase [41].

Because of the importance of Mcl-1 in cancer, we further studied the Mcl-1 stabilization that occurs upon TPA-induced ERK activation in BL41-3 cells. Our purpose was to better understand the finding that Thr 163 phosphorylation is associated with Mcl-1 stabilization in these and other cancer cells [42], but primes Mcl-1 for GSK3/phosphodegron-targeted degradation in normal fibroblasts [27]. Although phosphorylation at additional sites could be involved (e.g., Thr 92 [20]), this did not appear to be the case in BL41-3 cells [25]. The results of our studies showed that the GSK3-mediated pathway does not play a major role in Mcl-1 degradation in BL41-3 cells. This contrasts with what is observed in normal fibroblasts, but parallels the emerging findings showing that Mcl-1 degradation via this pathway is often impaired in cancer. The association of Thr 163 phosphorylation with Mcl-1 stabilization in this situation was recapitulated in transfected CHO cells, where Mcl-1 degradation was not affected by a non-phosphorylatable T163A mutation but was nearly completely blocked by a phosphomimetic T163E mutation. Along with ERK activation, Thr 163 phosphorylation, and Mcl-1 stabilization, TPA-treated BL41-3 cells exhibited markedly increased resistance to apoptosis-induction upon exposure to chemotherapeutic drugs. However, drug sensitivity was partially restored by an inhibitor of ERK activation. In contrast to normal cells where Thr 163 phosphorylation can promote GSK3/Ser 159 phosphodegron-targeted Mcl-1 degradation and death, in cancer cells in which Mcl-1 is not degraded through this pathway, ERK activation and Thr 163 phosphorylation are associated with reduced Mcl-1 degradation and striking drug resistance. Inhibition of ERK activation/Thr 163 phosphorylation represents a promising approach for promoting Mcl-1 degradation and drug sensitivity in these cancer cells.

## Methods

### Cell Lines and Treatments

BL41-3 cells were derived as a subline of BL41 Burkitt lymphoma cells as described [23], and were maintained in RPMI 1640 medium containing 7.5% FBS. The 5A-HSmyc CHO cell derivatives [43], [44] were maintained in alphaMEM medium containing 5% FBS. The mouse AKR-2B embryonic fibroblast line was from M. J. Getz (Mayo Foundation, Rochester, MN) [45], and was grown in McCoy’s 5A medium containing 5% FBS. TPA was from Alexis Biochemicals, LiCl, cycloheximide, LY294002, etoposide, vinblastine, cis-diamminedichloroplatinum(II) (cisplatin), Wortmannin, and cytosine arabinoside (Ara-C) were from Sigma, U0126 was from EMD Chemicals, and NaCl from Fisher Scientific.

### Mcl-1 Constructs and Transfection

Mcl-1 expression constructs were in the pcDNA 3.1 vector [25] containing a neo resistance marker. The WT-Mcl-1, Mcl-1-T163A, and Mcl-1-T162A constructs have been described, and Mcl-1-T163E and Mcl-1-S159A were prepared using the same methods [25], with the following primers. For MCL-1-T163E (upper primer) 5′- GGTCACTACCCTCGGAGCCGCCGCCAGCAG-3′ and (lower primer) 5′-CTGCTGGCGGCGGCTCCGAGGGTAGTGACC-3′, and for MCL-1-S159A (upper primer) 5′-CGGACGGGGCACTACCCTCGACGCCGCCGC-3′ and (lower primer) 5′-GCGGCGGCGTCGAGGGTAGTGCCCCGTCCG-3′. Transfection was carried out with Effectene (Qiagen) or Lipofectectamine 2000 (Invitrogen), using cells plated on the previous day.

### Western Analysis

Western blotting conditions that detect the human Mcl-1 protein have been described [25], [43], where no cross-reactivity with the Chinese hamster protein is seen. Standard size electrophoresis gels were used except as indicated where large format gels that separate the Mcl-1 doublet were used. For monitoring Mcl-1 decay in CHO cells, cells were replated to fresh medium on the day after transfection and exposed to CHX (20–25 micrograms/ml) 24 hours later [30]. For BL41-3 cells a concentration of 2.5 micrograms/ml CHX was used [25]. A ChemiDoc Molecular Imaging system (BioRad) became available and was used for some experiments, which allowed for estimation of changes in Mcl-1 expression. These changes were calculated as a decrease in Mcl-1 expression relative to parallel untreated control cells. While a useful antibody directed against Mcl-1 phosphoThr 163 is not available commercially, a small amount of the antibody described previously was available [46].

### Pulse/chase ^35^S-Met Labeling

Previously described methods were used [24]–[26]. In brief, one day after plating, cells were washed 3 times with and incubated in methionine-free RPMI medium containing 5% dialyzed FBS and 25 mM HEPES buffer. Cells were pulse-labeled with tran-^35^S-Met for 2 hours, and then chased with alphaMEM medium containing 5% FBS and an additional 15 mg/ml L-methionine. After harvesting and washing twice with PBS on ice, the pellet was lysed by passage through a syringe in lysis buffer [wash buffer (142.5 mM KCl, 5 mM MgCl_2_, 1 mM EGTA in 20 mM Tris-Cl, pH 7.4) containing 0.2% Nonidet P-40 and Sigma protease and phosphatase inhibitor cocktails]. The pellet was incubated overnight in the cold with antiMcl-1 antibody (Santa Cruz S-19) conjugated to Dynabeads (Dynal, Norway), washed twice with lysis buffer, once with wash buffer, and subjected to SDS polyacrylamide gel electrophoresis. The gel was fixed in 10% acetic acid: 30% methanol, soaked in NAAMP 100 Amplify (Amersham Biosciences, UK), dried, and exposed to X-ray film and a PhosphorImager screen, the latter being analyzed using ImageQuant software.

### Flow Cytometry

Flow cytometry was carried out using an FITC-conjugated antiMcl-1 antibody with the Caltag Fix&Perm kit (10,000 cells assayed for each sample).

### Cell Death Assays

Apoptotic cells as originally defined morphologically were scored using Wright’s Giemsa stained cytospin (Shandon) slide preparations [17], [47], [48]. PARP cleavage was assayed by Western blot analysis using the total PARP antibody (Cell Signaling), and chemiluminescent scanning of the membranes using the ChemiDoc system. Band intensities were quantified using the Fiji program (NIH ImageJ).

### Statistical Analysis

The half-life of decay of Mcl-1 encoded proteins (assayed using the ChemiDoc system) was estimated by non-linear regression using Prism 5 (GraphPad Software). Other statistical analyses were carried out using SigmaStat software.

## Results

### ERK Activation, Thr 163 Phosphorylation, and Stabilization of the Endogenous Mcl-1 Protein Occur Early after Exposure of BL41-3 Cells to TPA and are Subsequently Downregulated

BL41-3 cells exhibit abundant constitutive expression of endogenous Mcl-1 (∼5-fold higher than ML-1 cells stimulated with TPA [23]), and have proven very useful for studies of its posttranslational regulation. TPA-induced ERK activation results in little further increase in Mcl-1 expression in these cells (Fig. S1A, B), unlike in ML-1 and other cells [17], [21], [22], [25], [26]. BL41-3 cells are thus particularly useful for studies of TPA/ERK-induced Thr 163 phosphorylation [24], because effects on the stability of the protein can be examined in the absence of substantial **Mcl-1 induction**.

Our initial experiments further characterized the timing of the effects induced by TPA, since ERK activation is often transient [17], [49]. ERK activation and increased Thr 163 phosphorylation were detectable within 0.5 hours after TPA addition [24], [25] and maintained for about 6 hours, declining thereafter as monitored for up to 24 hours (Fig. 1B and Fig. S1A). To determine whether the decline in ERK activation and Thr 163 phosphorylation seen following prolonged TPA exposure would be reflected in a decline in Mcl-1 stabilization, we monitored Mcl-1 decay both immediately after TPA addition and 24 hours later. When CHX was applied to inhibit protein synthesis, Mcl-1 degradation was near-complete within 7 hours (Fig. 1C, upper left) but was slowed upon concomitant application of TPA (Fig. 1C, upper right), as in earlier studies [25]. However, when CHX was applied 24 hours after TPA, Mcl-1 stabilization was attenuated (Fig. 1C, lower left), although it could be restored by the application of additional TPA at this time (Fig. 1C, lower right). In sum, ERK activation, Thr 163 phosphorylation, and Mcl-1 stabilization occur as early events upon exposure of BL41-3 cells to TPA, and are all subsequently downregulated. These results reinforced previous findings, which had suggested a close association between these TPA-induced effects in that all were inhibited by the ERK pathway inhibitor U0126 ([25] and Fig. S1D).

### Mcl-1 Degradation in BL41-3 Cells is LiCl-insensitive

**Figure 2:**
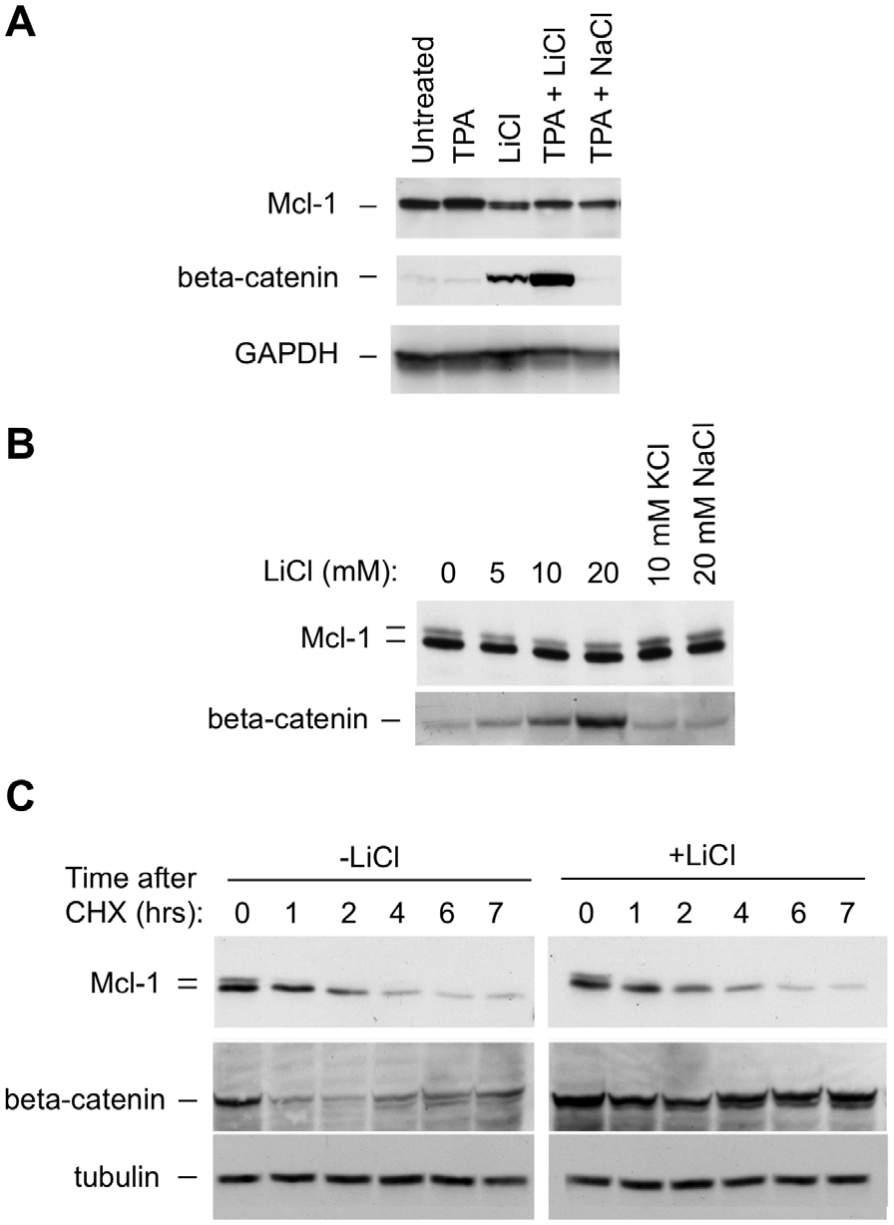
Mcl-1 degradation is insensitive to the GSK3 inhibitor LiCl in BL41-3 cells. **A:** BL41-3 cells were either left untreated or exposed to TPA (20 nM) and/or LiCl (20 mM, or NaCl as a control). After 18 hours, expression of Mcl-1, beta-catenin, and GAPDH and was assayed by Western blotting. Comparable results were observed when the cells were exposed to TPA for 30 minutes instead of 18 hours (not shown). **B:** BL41-3 cells were exposed to the indicated concentrations of LiCl (or KCl or NaCl as controls), and assayed for expression of Mcl-1 and beta-catenin after 24 hours. A large format gel that separates the Mcl-1 doublet bands was used. Slight inhibition of cell growth was seen at 10–20 mM LiCl (13 and 27%, respectively as compared to 5% with 20 mM NaCl). Results similar to those shown were obtained with an exposure time of 12 hours or a LiCl concentration of 40 mM (not shown). **C:** BL41-3 cells were incubated in the absence or presence of LiCl (20 mM) for 0.5 hours, at which time CHX was applied. Expression of Mcl-1, beta-catenin, and GAPDH was assayed after the indicated times. No substantial difference was seen in parallel cells exposed to NaCl (20 mM, not shown) instead of LiCl.

Mcl-1 can be targeted for degradation by GSK3, and exposure to TPA results in GSK3 inactivation in some cells [29], [31], [32], [50], [51]. It therefore seemed possible that TPA-induced GSK3 inactivation and inhibition of Mcl-1 degradation through this pathway might account for the stabilization seen in BL41-3 cells. As a means of examining this possibility, we probed for an effect of TPA on expression of the GSK3 target beta-catenin. The effect of the GSK3 inhibitor LiCl was monitored in parallel. The latter agent served as a positive control capable of causing an increase in beta-catenin expression (Fig. 2A lane 3, middle photograph). Our purpose was to determine whether TPA mimicked the effect of LiCl to produce an increase in beta-catenin expression, as this would be suggestive of an effect on GSK3. However, TPA did not appear to have such an effect in that it did not increase beta-catenin expression in the absence of LiCl (Fig. 2A lane 2, middle photograph).

Mcl-1 expression was also monitored in the above experiment, and was not found to be increased in the presence of LiCl (Fig. 2A, upper photograph). In fact, no change in Mcl-1 expression was seen even at LiCl concentrations that caused a maximal increase in beta-catenin expression and upon examination using large format gels (Fig. 2B). This observation was interesting as LiCl stabilizes Mcl-1 in a variety of cells and we had initially assumed that a GSK3-mediated, LiCl-sensitive pathway might be involved in BL41-3 cells. However, further observations were also consistent with the lack of a role for this pathway in Mcl-1 degradation in BL41-3 cells. Thus, exposure of these cells to Wortmannin or LY294002, PI3K inhibitors that can prevent inhibitory phosphorylation of GSK3 and thereby enhance the degradation of its targets, did not noticeably affect the expression of Mcl-1 while reducing that of beta-catenin (Fig. S2A). In addition, LiCl was not found to affect Mcl-1 degradation in the presence of CHX (Fig. 2C). In sum, Mcl-1 degradation in BL41-3 cells appeared to be largely LiCl-insensitive, and TPA did not act by mimicking the effect of this GSK3 inhibitor. These results, taken together with previous findings (Fig. 1 and [25]) suggested that Thr 163 phosphorylation might be associated with Mcl-1 stabilization in cells in which GSK3-targeted degradation does not play a major role. This point was considered further below, using the transfectable CHO cell system in which phosphorylation site mutants could be examined.

### Mcl-1 Degradation does not depend on the GSK3-targeted Phosphodegron and is Markedly Slowed by a T163E Mutation in Transfected CHO Cells

**Figure 3:**
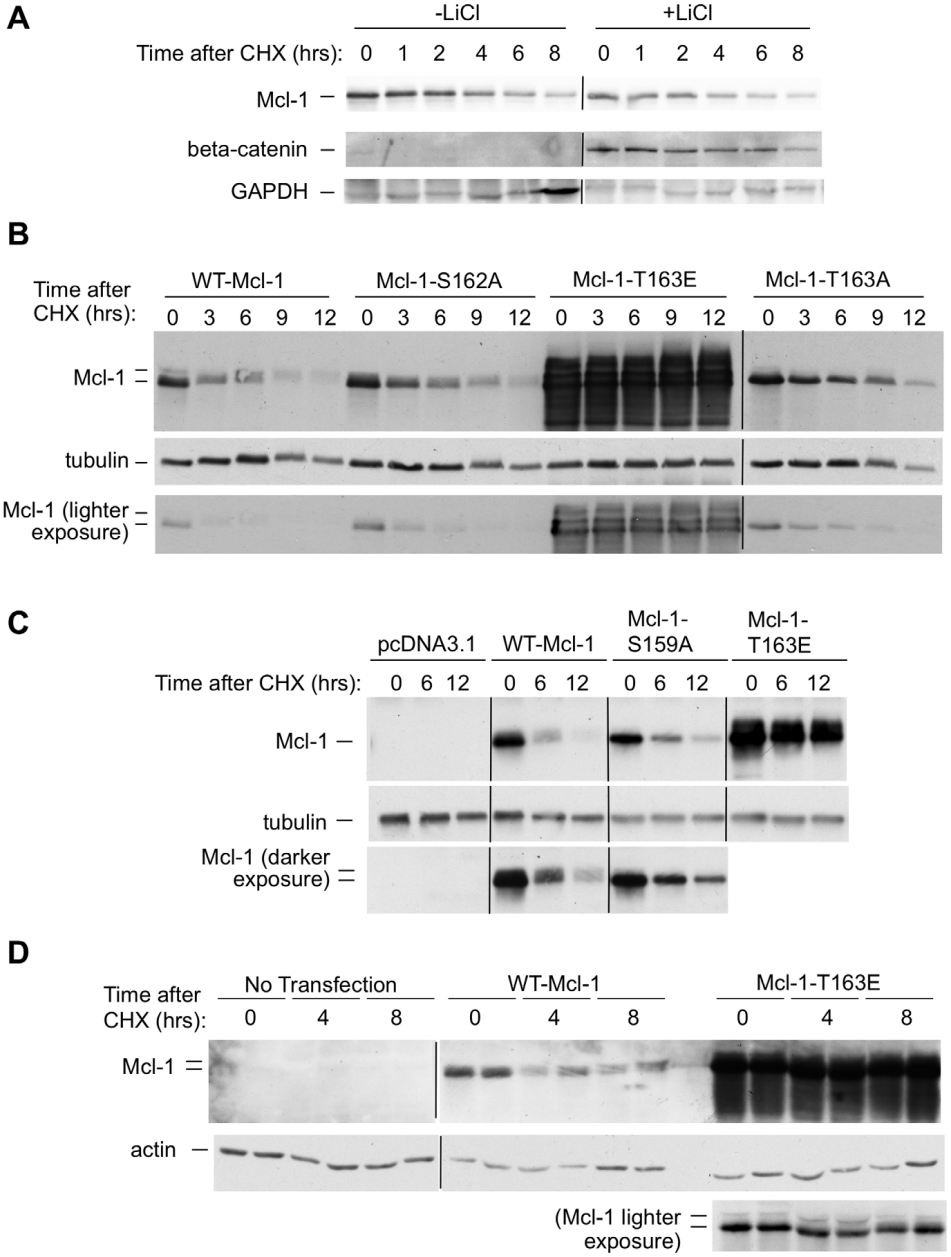
Mcl-1 degradation is slowed by a T163E mutation (but not T163A) in transfected CHO cells. **A:** CHO cells were transfected with WT-Mcl-1 and incubated in the presence of LiCl (+LiCl; 20 mM) or in its absence (−LiCl) where 20 mM NaCl was added in the latter case. After 10 hours, CHX was applied and expression of the introduced WT-Mcl-1 gene product, and endogenous beta-catenin and GAPDH, was assayed after the indicated times (ChemiDoc). The half-life of Mcl-1 decay was estimated to be ∼4 hours in cells exposed to LiCl, and 3.7 hours in the controls exposed to NaCl. No substantial difference was seen in parallel cells not exposed to NaCl or LiCl. The blot shown is representative of three independent experiments. **B–C:** CHO cells were transfected with the indicated constructs, replated on the following day, and incubated for a 24-hour period to allow expression. CHX was then added and expression of the introduced Mcl-1 gene product and endogenous beta-tubulin was assayed after the indicated times by Western blotting. In Panel B, the decline in Mcl-1 expression at 3 hours in 2 independent experiments ranged from 53–66% with WT-Mcl-1, 25–54% with Mcl-1-S162A, and 25–50% with Mcl-1-T163A. While the decline in expression with WT-Mcl-1 appeared be slightly greater than that seen with Mcl-1-T163A, this was not a consistent finding. A short autoradiographic exposure is also shown because additional bands were detected at the high levels of expression obtained with Mcl-1-T163E. At the end of the 12-hour observation period, expression of Mcl-1-T163E was decreased by ∼15% in Panel B and ∼27% in Panel C, as estimated from short autoradiographic exposures. **D:** AKR-2B cells transfected with the indicated constructs were replated on the following day, at which time cell viability (trypan blue dye exclusion) was 88–92% in untransfected as well as transfected cultures. One day later, cells were exposed to 25 micrograms/ml CHX and assayed after the indicated times for expression of the introduced Mcl-1 gene product or endogenous actin by Western blotting. Duplicate plates are shown in adjacent lanes.

CHO cells provide a readily transfectable system useful for studying mutations at sites found to undergo post-translational modification endogenously in BL41-3 cells [24], [25], [30], [43]. CHO cells contain basal activated ERK unlike BL41-3 cells. Accordingly, Thr 163 phosphorylation occurs upon transfection with WT-Mcl-1, and is not further increased by the addition of TPA [25]. We therefore set out to examine the effect of a non-phosphorylatable T163A mutation, as well as a phosphomimetic T163E mutation, upon transfection of mutant constructs into CHO cells. Interestingly, preliminary results showed that LiCl did not affect the expression or degradation of WT-Mcl-1 in CHO cells, although beta-catenin expression was increased (Fig. 3A and Fig. S2B). This provided a parallel to the findings in endogenously expressing BL41-3 cells (Fig. 2C), and suggested that Mcl-1 degradation might likewise not be mediated via GSK3 in transfected CHO cells. In this case, a T163A mutation would not be expected to affect Mcl-1 degradation. This would differ from what is seen in normal fibroblasts, where a T163A mutation slows Mcl-1 degradation because Thr 163 phosphorylation primes GSK3-induced Ser 159 phosphorylation and degradation [27]. Indeed, the Mcl-1-T163A-encoded protein underwent rapid degradation upon exposure of transfected CHO cells to CHX (Fig. 3B), as did the WT-Mcl-1-, Mcl-1-S162A- and Mcl-1-S159A-encoded proteins (Fig. 3B, C). Mcl-1-S162A represents an additional control as phosphorylation has not been observed at this site [25]. We note that, as in previous reports [27], an S159A/T163A double mutant was not examined, because a near-complete loss of Mcl-1 phosphorylation is seen with the T163A mutation in CHO cells [25] and this priming site mutation prevents Ser 159 phosphorylation in fibroblasts [27]. Taken together, these results with non-phosphorylatable mutations as well as LiCl suggest that the GSK3-targeted phosphodegron does not play a major role in Mcl-1 degradation in transfected CHO cells. In this situation, the Mcl-1-T163E encoded protein exhibited striking stabilization [Fig. 3B (see lighter exposure included at the bottom because of the extensive stabilization seen with this construct) and Fig. 3C]. Similar results were obtained with AKR-2B cells (Fig. 3D).

### Mcl-1-T163E Exhibits Elevated Accumulation and Prevents the Outgrowth of Stable G418-resistant Transfectants

**Figure 4:**
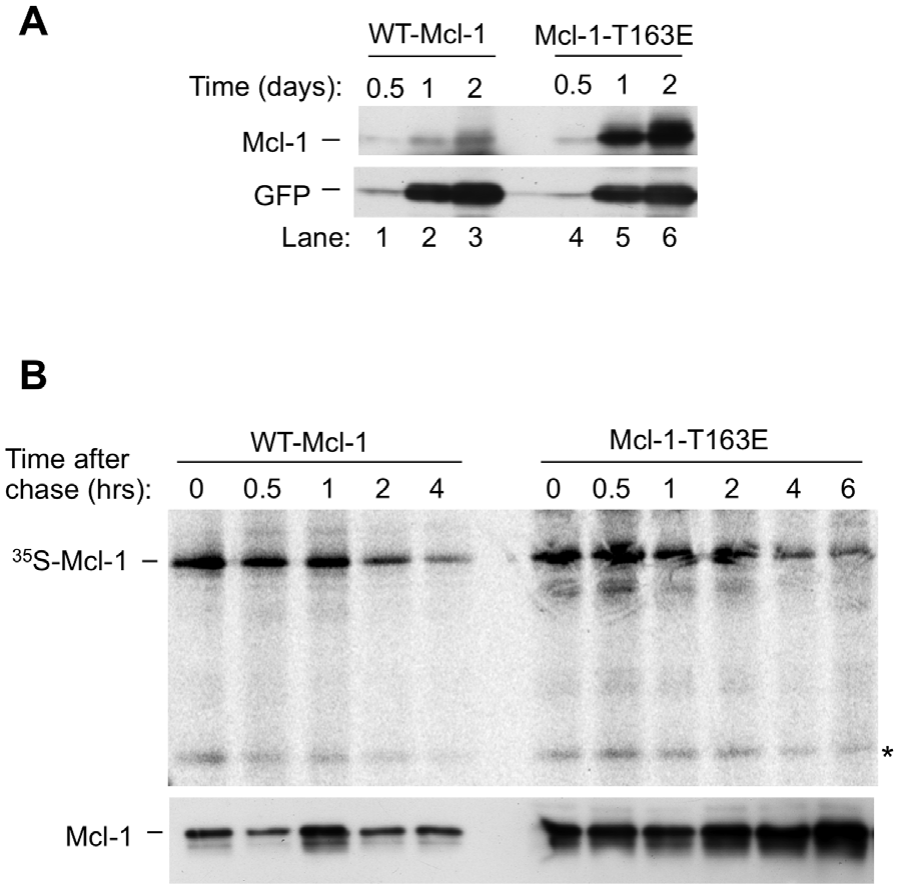
Slowed degradation without increased synthesis increases Mcl-1 accumulation in the presence of the T163E mutation. **A:** CHO cells were co-transfected with either WT-Mcl-1 or Mcl-1-T163E along with pEGFP, and assayed at the indicated times for expression of the introduced Mcl-1 gene product and EGFP by Western blotting. The rate of increase of the Mcl-1-T163E protein was estimated to be at least 10 times greater than that of WT-Mcl-1. Approximately equivalent expression of EGFP in WT-Mcl-1- and Mcl-1-T163E-transfected cultures was also seen upon assay by flow cytometry (not shown). **B:** CHO cells were transfected with WT-Mcl-1 or Mcl-1-T163E and subjected to a 2-hour pulse exposure to ^35^SMet, at which time a “time 0” sample was harvested. A chase with medium containing non-radioactive methionine was then performed and the ^35^SMet- labeled Mcl-1 proteins were assayed after the indicated times (upper photograph). A non-specific band of ∼32 kd is included on the figure (*) for comparative purposes and a separate aliquot of each sample was assayed for total Mcl-1 expression by Western blotting (lower photograph). The half-life of decay was estimated by PhosphorImager to be ,2.5-fold longer with ^35^S-Met- Mcl-1-T163E than with ^35^S-Met-WT-Mcl-1.

In the above experiment, Mcl-1-T163E exhibited elevated accumulation during the expression period prior to the application of CHX (Fig. 3B, time 0). Examination of the time course of accumulation confirmed that a dramatic increase occurred after transfection with Mcl-1-T163E as compared to WT-Mcl-1 (Fig. 4A, lanes 4–5 versus lanes 1–2).

Upon pulse/chase metabolic labeling, ^35^S-Met-Mcl-1-T163E and ^35^S-Met-WT-Mcl-1 demonstrated equivalent synthesis during the initial pulse (Fig. 4B, time 0). After the chase, the decay of ^35^S-Met-Mcl-1-T163E was slowed as compared to that of ^35^S-Met-WT-Mcl-1 (Fig. 4B), although slowing here was not as marked as had been seen upon monitoring the total Mcl-1-T163E protein by Western blotting (Fig. 3B). This could reflect the fact that pulse/chase labeling assays the newly synthesized protein (generally a fraction of the total), or other differences between these methods [52], [53]. Taken as a whole, the above findings reinforced and extended those in BL41-3 cells, showing that the T163E mutation resulted in Mcl-1 stabilization in CHO cells, where degradation was largely independent of the GSK3-targeted phosphodegron at S_159_LPST_163_P.

The extensive stability and elevated accumulation seen with Mcl-1-T163E may relate to a further observation, which was that we were unable to derive stably transfected cell lines with this construct. This observation was initially somewhat unexpected, because continuously growing transfected cell lines were previously obtained with WT-Mcl-1 upon selection with G418 (due to the neo^R^ marker [43]) and could also be obtained with Mcl-1-T163A. Mcl-1 expression in these stably transfected cell lines was in the range seen in cells that express Mcl-1 endogenously, such as BL41-3 cells and TPA-treated ML-1 cells ([43] and upper panel and legend).

In view of the above observation, we examined Mcl-1-T163E-transfected cultures at early times after transfection and selection with G418. Directly after transfection, a range of Mcl-1 expression levels was observed (Fig. S3A lower panel). When G418 was then applied to eliminate untransfected cells, viable cells did not grow out from Mcl-1-T163E-transfected cultures, unlike what occurred with WT-Mcl-1 [Fig. S3B (filled symbols in right versus middle panel), where cells grew in the absence of G418 in both cases but lost Mcl-1 expression (open symbols)]. The G418-resistant cells that grew out with WT-Mcl-1 exhibited much lower levels of expression than the initial bulk transfected population (Fig. S3C, lanes 10–12). In contrast, abundant expression was maintained in Mcl-1-T163E-transfected cultures (Fig. S3C, lanes 16–18). Overall, selection with G418 resulted in the outgrowth of transfectant lines exhibiting expression in the endogenous range with WT-Mcl-1 as well as Mcl-1-T163A, but not with Mcl-1-T163E.

In addition to its antiapoptotic effect, Mcl-1 has been reported to inhibit cell proliferation in some systems [54], [55]. This effect was not observed in stable transfectants expressing the protein at levels in the endogenous range [56]. However, inhibition of proliferation has been seen upon transient transfection or other approaches capable of producing high levels of expression. The fact that the WT-Mcl-1-transfected CHO cell lines exhibited expression in the endogenous range (Fig. S3A upper panel) suggests that such cells may have had a growth advantage over those with higher levels of expression. Stable transfectants with another recipient line likewise exhibited expression in the endogenous range, but not higher [56]. Overall, while WT-Mcl-1 promotes viability in stably transfected clones expressing levels in the endogenous range, it remains to be determined whether higher expression levels result in additional effects such as inhibition of cell proliferation.

In view of the above considerations, it is perhaps not surprising that stably transfected lines were not obtained with Mcl-1-T163E. We note that expression of endogenous tubulin was reduced in cultures transfected with this construct (Fig. S3C), which could relate to elevated expression of the transfected protein and/or the presence of the non-removable Glu mutation. We also note that some cells within the initial bulk Mcl-1-T163E-transfected population exhibited lower expression (Fig. S3A lower panel). Since the Mcl-1-T163E protein accumulates rapidly (Fig. 4A), it is possible that subsequent ongoing accumulation prevented the outgrowth of these cells.

We have previously examined the effect of WT-Mcl-1 in cells that were selected as stable transfectants in G418, and then derived as clonal transfectant lines [43]. Expression of exogenously introduced WT-Mcl-1, at levels comparable to those seen endogenously, resulted in moderate viability-enhancement upon exposure to apoptosis-inducing stimuli. With this moderate effect and the variability seen with non-clonal and/or transient transfectants, obtaining stable transfectant clones was important for obtaining meaningful results in terms of effects on viability. The inability to obtain stable transfectants with Mcl-1-T163E thus precluded meaningful study of its effect on cell viability, a limitation that will be tackled in the future by deriving inducible transfectants. In sum, elevated expression was an expected finding given the stability of Mcl-1-T163E, but prevented the outgrowth of stable transfectants in which the effects of this mutation could be further examined. Since elevated accumulation of Mcl-1-T163E could not be prevented in transfected CHO cells, the effect of Thr 163 phosphorylation was further examined in BL41-3 cells exposed to TPA.

### TPA-induced ERK Activation and Thr 163 Phosphorylation are Associated with the Maintenance of Mcl-1 Expression and Increased Chemotherapeutic Drug Resistance in BL41-3 Cells

**Figure 5:**
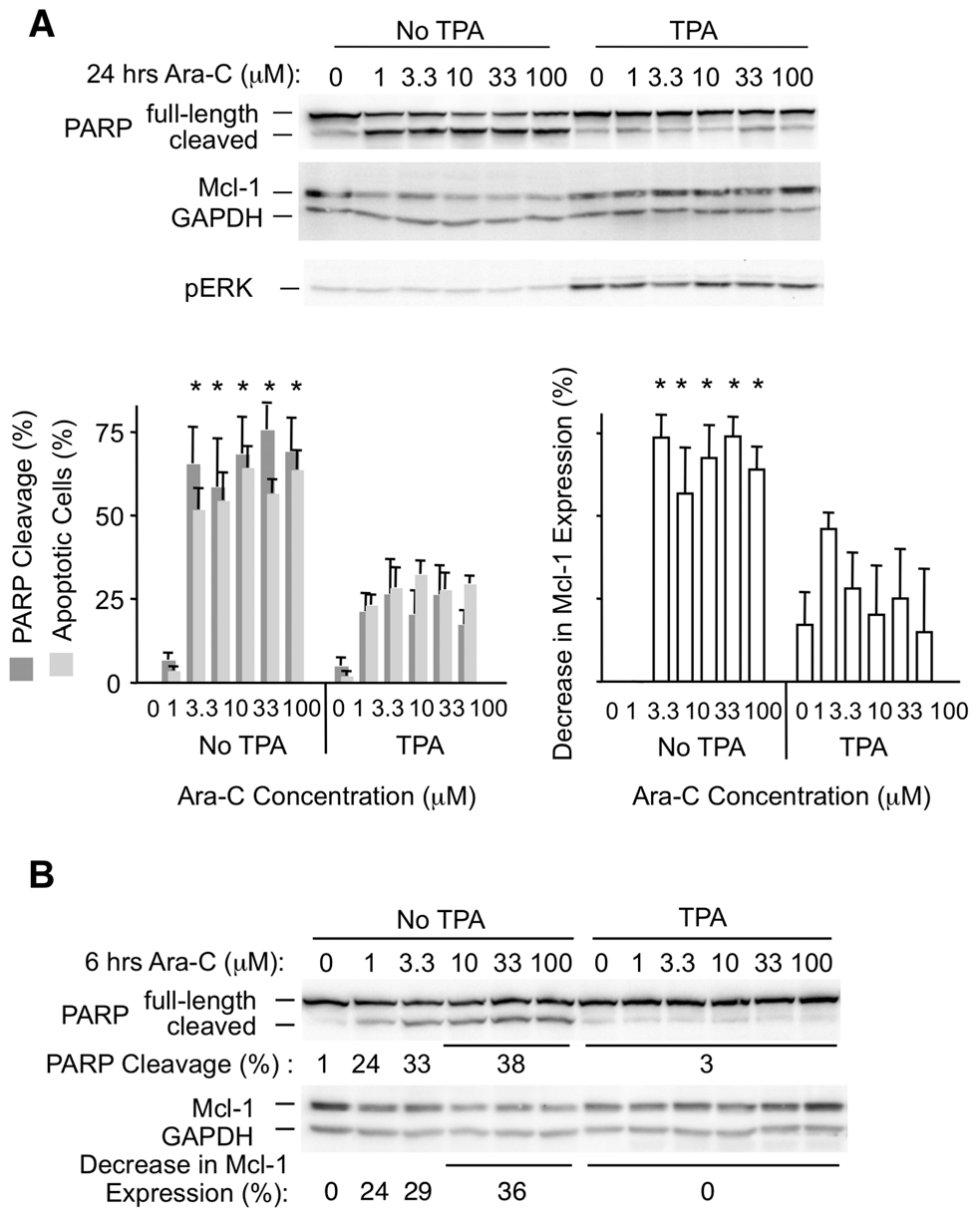
Ara-C-induced Mcl-1 degradation and cell death are inhibited upon TPA-induced ERK activation in BL41-3 cells. **A:** BL41-3 cells were incubated in the absence or presence of TPA (5 nM) for 0.5 hours, followed by the addition of the indicated concentrations of Ara-C. After 24 hours, PARP cleavage and Mcl-1 expression were assayed (ChemiDoc). A representative experiment is included (upper photograph) along with the average (± SE) of 5 independent experiments. Also shown for comparative purposes is the percentage of cells exhibiting morphological apoptosis, assayed with a concentration of 1 nM TPA applied 1 hour before the addition of Ara-C (mean of 3 independent experiments ± SE run in parallel with previous studies; some of the BL41-3 cell samples not treated with TPA were included in a previous publication comparing these to BL41 parental cells (Fig. 4 in reference [23]). *significant difference in the presence of Ara-C as compared to its absence (p<0.05; ANOVA, post-hoc Holm-Sidak test). **B:** BL41-3 cells were incubated in the absence or presence of TPA (5 nM) for 0.5 hours, followed by the addition of the indicated concentrations of Ara-C. After 6 hours, PARP cleavage and Mcl-1 expression were assayed as in Panel A. The average of 4 experiments is shown below representative blots, where the values for Ara-C concentrations that had similar effects are shown together and overlined. For PARP Cleavage, the SE of the values shown in successive lanes was 1, 8, 7, 3, and 3, respectively. The Decrease in Mcl-1 Expression was calculated relative to untreated control cells; the SE of the values shown (other than the untreated control value) was 11, 8, 8, and 7. Linear regression analysis of the Decrease in Mcl-1 Expression versus PARP Cleavage for 0–10 micromolar Ara-C in the absence of TPA demonstrated an average slope of 1.2 [±0.49 (SE) for the 4 experiments)], with an average r^2^ value of 0.87.

Since reduced degradation of Mcl-1 is frequently associated with drug resistance [33], [34], [57], we tested for such effects in BL41-3 cells exposed TPA. This was first examined using Ara-C, an inhibitor of DNA synthesis previously found to induce morphological apoptosis in BL41-3 cells treated for 24 hours [23]. This treatment produced morphological apoptosis in about 60% of cells, along with ∼60% Poly ADP ribose polymerase (PARP) cleavage and a decrease in Mcl-1 expression of ∼60% (Fig. 5A left). Similar effects were seen with Ara-C concentrations of 1–100 micromolar, which may relate to the fact that this agent acts on cells in S-phase of the cell cycle. These effects were strikingly reduced (to values of ∼20%; Fig. 5A right) when Ara-C was applied in the presence of TPA to induce ERK activation and Thr 163 phosphorylation. Lower concentrations of Ara-C by itself resulted in lesser effects on PARP cleavage as well as Mcl-1 expression (Fig. S4A). Here, PARP cleavage with 0.1 micromolar Ara-C in the absence of TPA was approximately equivalent to that seen with a 10- to 100-fold higher concentration of Ara-C in the presence of TPA. In sum, the maintenance of Mcl-1 expression in the presence of TPA was associated with a ≥10-fold increase in resistance to the chemotherapeutic agent.

Morphological apoptosis is seen in BL41-3 cells exposed to Ara-C for 24 hours but is not readily apparent at 6 hours [23]. However, PARP cleavage can often be detected at early times. Indeed, exposure to Ara-C by itself for 6 hours resulted in an increase in PARP cleavage along with a decrease in Mcl-1 expression (Fig. 5B). There was a close correspondence between these two effects in that the magnitude of the increase in PARP cleavage mirrored the decrease in Mcl-1 expression (Fig. 5B and legend). Since both effects were in evidence at this early time point, it could not be distinguished whether one or the other might represent a primary event. In any case, increased apoptosis and decreased Mcl-1 expression occurred in tandem with a variety of Ara-C concentrations and at exposure times of 6 or 24 hours, and both effects were strongly suppressed upon concomitant exposure to TPA.

**Figure 6:**
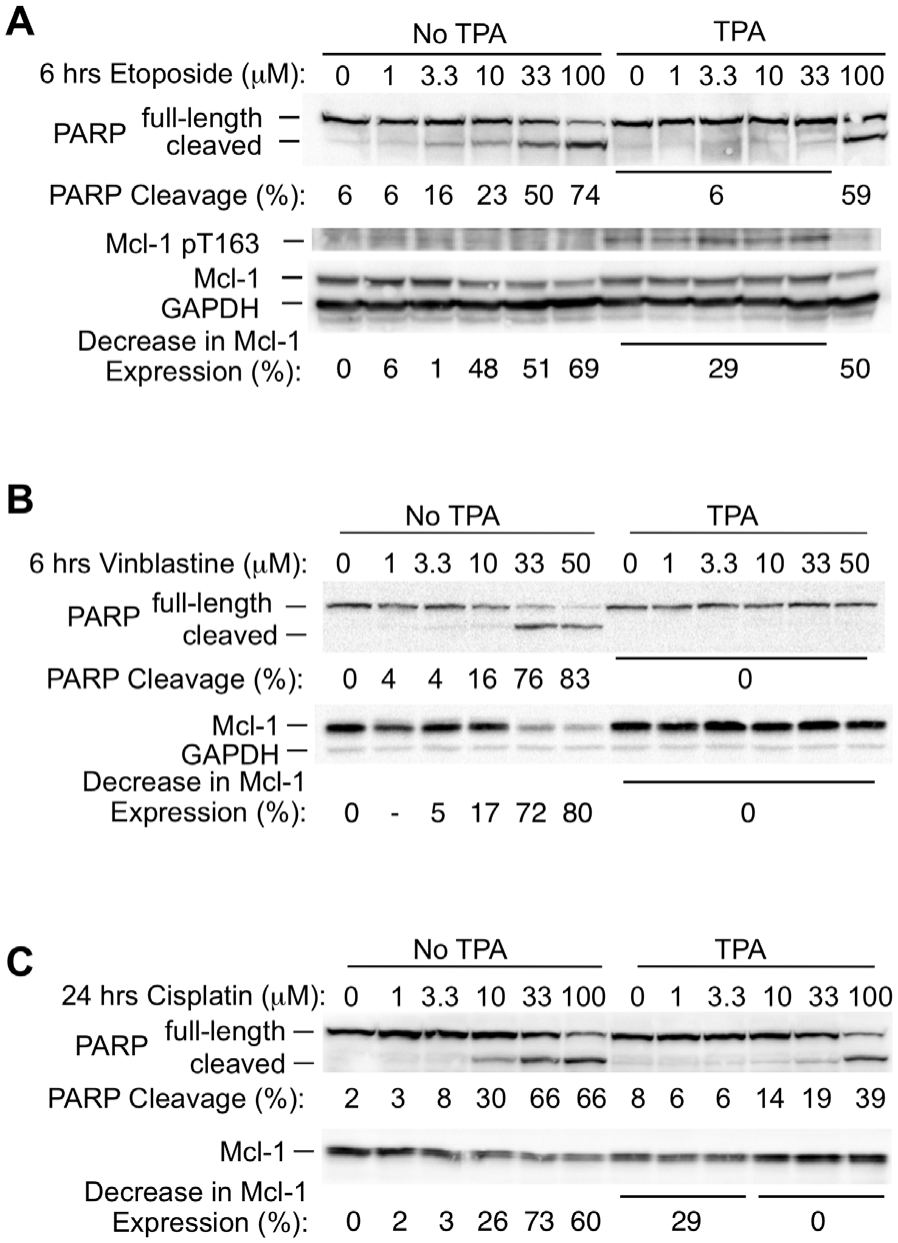
TPA-induced Mcl-1 stabilization is associated with increased resistance to various chemotherapeutic agents in BL41-3 cells. **A:** BL41-3 cells were incubated in the absence or presence of TPA (5 nM) for 0.5 hours, followed by the addition of the indicated concentrations of etoposide. After 6 hours, PARP cleavage, Mcl-1 expression, and Thr 163 phosphorylation were assayed (ChemiDoc), where the values for PARP Cleavage (%) and the Decrease in Mcl-1 Expression (%) are indicated below the respective blots. **B:** BL41-3 cells were incubated in the absence or presence of TPA (5 nM) for 0.5 hours prior to the addition of the indicated concentrations of vinblastine. After 6 hours, PARP cleavage and Mcl-1 expression were assayed as in Panel A. **C:** BL41-3 cells were incubated in the absence or presence of TPA (5 nM) for 0.5 hours prior to the addition of the indicated concentrations of cisplatin. After 24 hours, PARP cleavage and Mcl-1 expression were assayed as in Panel A. The blot shown is representative of 3 independent experiments. The SE of the values shown in successive lanes for PARP Cleavage was 2, 2, 5, 11, 17, 0.2, 4, 2, 3, 4, 6, and 13, respectively. The Decrease in Mcl-1 Expression was calculated relative to untreated control cells; the SE of the other values shown was 10, 12, 18, 12, 7, 9, and 11.

We next tested additional chemotherapeutic agents, since Mcl-1 can broadly inhibit the induction of apoptosis [56]. Exposure to the topoisomerase II inhibitor etoposide, which was previously found to induce morphologic apoptosis in BL41-3 cells [23], resulted in PARP cleavage and decreased Mcl-1 expression at 6 hours (Fig. 6A). Both these effects were inhibited in the presence of TPA. Interestingly, TPA-induced protection from apoptosis, as well as its effect on Mcl-1 stabilization, were overridden at an elevated concentration of etoposide (100 micromolar; Fig. 6A, last lane). Under these conditions, TPA-induced Thr 163 phosphorylation was not in evidence, and PARP cleavage occurred along with a decrease in Mcl-1 expression.

We also tested the microtubule-disrupting agent vinblastine, which affects cells in G2/M phase of the cell cycle. Vinblastine can also cause rapid death (e.g., within hours in ML-1 cells), which is inhibited in the presence of ERK activation [49]. Exposure of BL41-3 cells to vinblastine alone for 6 hours resulted in up to ∼80% PARP cleavage and an ∼80% decrease in Mcl-1 expression (Fig. 6B). These effects were essentially completely blocked in the presence TPA. In sum, chemotherapeutic drugs having widely varying modes of action caused a decline in Mcl-1 expression in conjunction with the induction of apoptosis, where concomitant exposure to TPA suppressed this decline and was associated with pronounced drug resistance.

In the above experiment with vinblastine, little or no effect was seen upon 6 hours of exposure in the presence of TPA. This recalled observations with Ara-C at 6 hours (Fig. 5B), where the effects of Ara-C were also suppressed by TPA upon 24 hours of exposure (Fig. 5A). Upon treatment with vinblastine for 24 hours, PARP cleavage and decreased Mcl-1 expression occurred in the presence of TPA, but were less marked than in the absence of TPA (PARP cleavage of ∼68% versus >85%, respectively; Fig. S4B). We also examined PARP cleavage and Mcl-1 expression after 24 hours of exposure to cis-diamminedichloroplatinum(II) (cisplatin), a DNA-crosslinking agent that acts on cells in the various phases of the cell cycle. The presence of TPA resulted in inhibition of PARP cleavage and the maintenance of Mcl-1 expression (Fig. 6C). These observations were interesting in light of the above finding that, when TPA is applied by itself, ERK activation, Thr 163 phosphorylation and Mcl-1 stabilization are in decline at 24 hours (Fig. 1). When TPA is applied in the presence of chemotherapeutic drugs, it is possible that the maintenance of Mcl-1 expression and protection from apoptosis at early times (Fig. 5B, and Fig. 6A, B) means that more cells remain viable upon prolonged exposure.

### U0126 Partially Restores Chemotherapeutic Drug Sensitivity in TPA-treated BL41-3 Cells

**Figure 7:**
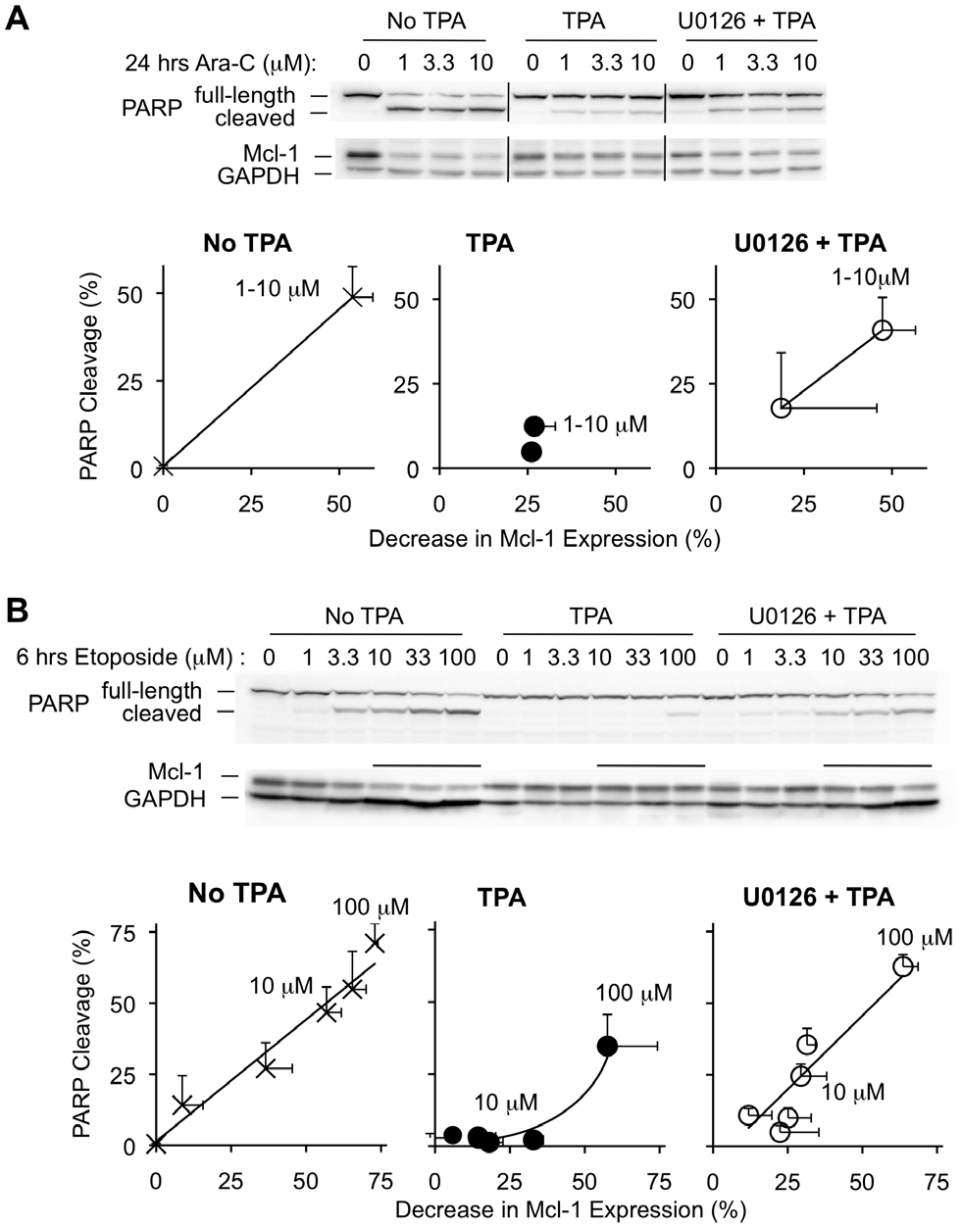
Inhibition of TPA-induced ERK activation partially restores chemotherapy-induced Mcl-1 degradation and cell death. **A:** BL41-3 cells were exposed to U0126 (25 micromolar) for 0.5 hours, followed by the addition of TPA (5 nM) for 0.5 hours and then addition of the indicated concentrations of Ara-C. After 24 hours, PARP cleavage and Mcl-1 expression were assayed (ChemiDoc). The blot shown is representative of 3 independent experiments, where similar effects were seen with the various Ara-C concentrations as above (Fig. 5A). In the graph below the blot, the Decrease in Mcl-1 Expression (±SE) is plotted against PARP Cleavage (±SE), where points representing the average of cells treated with 1–10 mM Ara-C are labeled and the unlabeled points represent cells not treated with Ara-C. **B:** BL41-3 cells were exposed to U0126 (25 micromolar) for 0.5 hours, followed by the addition of TPA (5 nM) for 0.5 hours and then addition of the indicated concentrations of etoposide. After 6 hours, PARP cleavage and Mcl-1 expression were assayed. The blot shown is representative of 3 independent experiments. The lanes that are overlined on the Mcl-1 blot represent concentrations of etoposide where U0126 resulted in partial inhibition of Mcl-1 stabilization. In the graph, the average Decrease in Mcl-1 Expression for each concentration of etoposide (±SE) is plotted against average PARP Cleavage (±SE). The lines shown for No TPA and U0126+ TPA were determined by linear regression, where the points representing 10 and 100 micromolar etoposide are labeled. The slope of this line averaged 0.85 in the absence of TPA [±0.1 (SE); average r^2^ of 0.92], and 1.0 in the presence of U0126+ TPA (±0.19; average r^2^ of 0.7).

In a final series of experiments, we preincubated BL41-3 cells with the ERK pathway inhibitor U0126, prior to exposure to TPA and chemotherapeutic agents. Our rationale was that U0126 might be expected to inhibit TPA-induced Mcl-1 stabilization [25], thereby allowing us to determine whether drug sensitivity and Mcl-1 degradation could be restored. This was first examined in the presence of Ara-C where PARP cleavage induced by this agent (∼50%) is graphed against the decrease Mcl-1 expression (∼50%; Fig. 7A left). These effects were inhibited by TPA, and partially restored when U0126 was added prior to TPA (to values of ∼45%; Fig. 7A right).

We also examined the effects of increasing concentrations of etoposide, both to extend the findings above (Fig. 5A) and to assess the effect of U0126. With etoposide by itself, PARP cleavage and decreased Mcl-1 expression occurred approximately in proportion to each other (Fig. 7B left). The presence of TPA resulted in a >10-fold increase in resistance to PARP cleavage: whereas a concentration of 10 micromolar etoposide caused ∼50% PARP cleavage by itself, essentially no effect was seen in the presence of TPA and, in fact, 100 micromolar etoposide caused only ∼33% PARP cleavage (Fig. 7B middle). When U0126 was added prior to TPA, the effects of etoposide on PARP cleavage and Mcl-1 expression were partially restored. Here, a concentration of 10 micromolar etoposide caused ∼25% PARP cleavage and an ∼25% decrease in Mcl-1 expression, these values being ∼60% with 100 micromolar etoposide. Since both effects were partially restored, the relationship between Mcl-1 expression and PARP cleavage in the presence of U0126 plus TPA was virtually identical to that seen in cells not treated with these agents (i.e., the curves in Fig. 7B left and right have similar slopes). Overall, to the extent that TPA-induced maintenance of Mcl-1 expression could be inhibited by U0126, apoptosis induced by etoposide or Ara-C could be restored.

## Discussion

Mcl-1 is normally subject to rapid up- and downregulation, to modulate cell viability in response to environmental signals and maintain tissue homeostasis [1]. Mcl-1 can be targeted for degradation by several pathways, some of which are regulated post-translationally. Phosphorylation at Thr 163 was initially observed in TPA-treated BL41-3 cells, where it is associated with Mcl-1 stabilization [25]. Thr 163 phosphorylation also occurs in UV-irradiated mouse fibroblasts where it primes for GSK3-induced Ser 159 phosphorylation and Mcl-1 degradation [27]. In, the present work, this pathway was not found to play a major role in Mcl-1 degradation in BL41-3 cells. These cells may thus provide a model for the many types of cancer cells that exhibit impaired Mcl-1 degradation through the GSK3-targeted pathway [20], [32]. The finding that Thr 163 phosphorylation can promote Mcl-1 stabilization in this situation in BL41-3 cells was mimicked in CHO cells transfected with phosphorylation site mutants. Overall, Thr 163 phosphorylation can prime GSK3-targeted Mcl-1 degradation to promote death in normal cells; however, in cancer cells in which degradation is not dependent on this pathway, ERK activation and the induction of Thr 163 phosphorylation are associated with marked Mcl-1 stabilization and chemotherapeutic drug resistance.

Whereas Thr 163 phosphorylation in BL41-3 cells is induced by ERK, the priming phosphorylation in mouse fibroblasts is carried out by JNK, activated upon UV irradiation. Therefore, additional events induced by JNK may influence GSK3/phosphoSer 159 mediated Mcl-1 degradation in this system. Further studies of non-transformed hematopoietic cell lines may be informative in this regard. In the latter, Thr 163 phosphorylation is thought to prime the GSK3-induced Ser 159 phosphorylation/Mcl-1 degradation that occurs following growth factor deprivation [31]. However, whether ERK or JNK is involved has not yet been investigated. Taken together, findings from various systems suggest that MAP kinase (ERK or JNK)-induced phosphorylation at Thr 163 can either prime for Mcl-1 degradation – in cells in which this is targeted by GSK3-induced Ser 159 phosphorylation – or have the opposite effect to stabilize Mcl-1 in cells in which degradation is carried out by other, GSK3-independent pathways.

Abundant, dysregulated Mcl-1 expression is an important determinant of drug resistance in cancer [8], [11], [20], [32], [39], [58]. This often involves alterations affecting GSK3-targeted Mcl-1 degradation, such as GSK3 inactivation or changes in downstream components in the pathway [13], [28], [32]–[34], [39]–[41]. However, it is not clear why reduced Mcl-1 degradation through one pathway provides a substantial advantage to tumor cells, since multiple alternative pathways can target Mcl-1 degradation and might be expected to provide at least partial compensation. In the case of inactivation of the F-box protein FBW7, for example, Mcl-1 degradation is slowed but still occurs relatively rapidly [33], [34]. The present findings provide food for thought in this regard as they show that – in cells in which Mcl-1 degradation does not depend on the GSK3/phosphodegron – another common event in cancer, ERK activation, is associated with Thr 163 phosphorylation, Mcl-1 stabilization, and a dramatic increase in drug resistance. These findings also point to the importance of future studies aimed at assessing whether Mcl-1 Thr 163 phosphorylation could serve as a resistance marker in cancer patients.

The various events induced by TPA in BL41-3 cells appear to be closely linked. TPA-induced ERK activation, Thr 163 phosphorylation, and Mcl-1 stabilization occur as early, U126-inhibitable events and are subsequently downregulated. Similarly, upon exposure to chemotherapeutic drugs, TPA-induced inhibition of Mcl-1 degradation and apoptosis are seen at early times and exhibit a close correspondence. Because of this, a cause/effect relationship between these events could not be distinguished. Likewise, causality could not be meaningfully addressed via transfection with Mcl-1-T163E, because the extensive stability of the mutant protein resulted in its build-up to levels that far exceeded those seen physiologically. We also note that TPA has a multitude of effects, and other effects besides ERK activation, Thr 163 phosphorylation, and Mcl-1 stabilization may contribute to the drug resistance observed in BL41-3 cells. Whatever the case, a variety of chemotherapeutic drugs, applied at a range of doses, uniformly caused a decrease in Mcl-1 expression that correlated with the increase in PARP cleavage (approximately a 1∶1 correlation), both events being inhibited upon TPA-induced ERK activation and Thr 163 phosphorylation. Thus, effects on Mcl-1 expression may contribute to, and serve as a surrogate marker for, effects on drug sensitivity/resistance.

A further observation related to those above came out of studies of TPA-induced viability-protection in cells treated with chemotherapeutic agents for 24 hours. While the direct effects of TPA on ERK activation, Thr 163 phosphorylation, and Mcl-1 stabilization – in the absence of chemotherapeutic drugs - were downregulated at this time, cell exposed to chemotherapeutic agents for 24 hours exhibited less apoptosis in the presence (versus the absence) of TPA. The maintenance of Mcl-1 expression and increased survival at early times may allow more cells to remain viable upon prolonged exposure. In terms of patient cancers in which GSK3-mediated Mcl-1 degradation is inactive, it remains to be determined whether the presence of ERK activation/Thr 163 phosphorylation contributes to resistance upon prolonged drug exposure.

It was also interesting that U0126 partially reversed the protective effects of TPA. Why reversal was only partial is not clear, since the inhibitor has been found to be highly effective in terms of minimizing TPA-induced ERK activation. One possibility is that other ERK-independent effects of TPA play a role, and another possibility is that some ERK activation occurs in the presence of U0126. Examination of pERK expression did not show detectable ERK activation in the presence of U1026 plus TPA, as in previous studies [25]. However, trace pERK appeared to be visible in the presence of these two agents plus etoposide (not shown). In previous studies, we have observed that a modicum of ERK activation, even if it is transient, can have a noticeable effect [49]. TPA is a powerful ERK inducer and it remains to be determined whether some activation, possibly transient, occurs in the present system. U0126 did not substantially counteract TPA-induced resistance at a low concentration of etoposide (3.3 micromolar), but promoted apoptosis in the presence of higher etoposide concentrations. In other words, in cancer cells exhibiting drug resistance in the presence of ERK activation and GSK3 inactivation, an effective approach may involve inhibition of the effects of ERK along with the application of full doses of chemotherapeutic agents.

Mcl-1 is highly regulated at multiple levels, and ERK-induced transcriptional and post-translational mechanisms have been suggested to act coordinately to rapidly increase or decrease expression [1]. The present findings lead us to speculate that coordination may also exist for pathways that degrade the protein. For example, when Mcl-1 degradation via the GSK3-targeted phosphodegron is downregulated, such as upon growth factor-stimulation [31], ERK-induced Thr 163 phosphorylation may provide a means for preventing degradation via alternative routes. This could avert a situation in which the GSK3-targeted pathway is downregulated, but this does not have a strong impact because other degradation pathways remain fully operational. In addition, as the growth stimulus wanes and GSK3 activation increases, the presence of Thr 163 phosphorylation may prime for ensuing Mcl-1 degradation. In other words, this could also provide a mechanism for preventing overly sustained Mcl-1 expression by coupling growth factor-induced Mcl-1 stabilization to priming for subsequent turnover. The interplay between these factors may then be exploited by cancer cells, where activation of ERK and inactivation of GSK3/phosphodegron-mediated degradation would promote extended Mcl-1 stabilization and drug resistance.

## Supporting Information

Figure S1 Exposure of BL41-3 cells to TPA to activate ERK stimulates Thr 163 phosphorylation in a fashion that is U0126-inhibitable but does not involve a substantial increase in Mcl-1 expression. **A:** BL41-3 cells were treated with 5 nM TPA and assayed for expression of Thr 163 phosphorylated Mcl-1 (Mcl-1 pT163), total Mcl-1, GAPDH, and phospho-ERK at the indicated times by Western blotting (ChemiDoc). **B:** BL41-3 cells were treated with 5 nM TPA and assayed for Mcl-1 expression using a large format gel that separates the 42/40 kd Mcl-1 doublet bands. **C:** BL41-3 cells were treated with the indicated concentrations of TPA and assayed for expression of Thr 163 phosphorylated Mcl-1, total Mcl-1, and GAPDH after 1 hour as in Panel A. **D:** BL41-3 cells were incubated in the absence or presence of U0126 (25 mM) for 30 minutes, and TPA (5 nM) was then added as indicated for an additional 3 hours. Expression of Thr 163 phosphorylated Mcl-1, total Mcl-1 and GAPDH was assessed as in Panel A. The asterisk indicates a non-specific band.
(TIF)

Figure S2 LiCl does not affect Mcl-1 expression in BL41-3 cells or WT-Mcl-1-transfected CHO cells. **A:** BL41-3 cells were either left untreated or exposed to Wortmannin (1 µM) or LiCl (20 mM) for 18 hours, or to LY294002 (20 µM) for 3 hours, and then assayed for the expression of Mcl-1, GAPDH, GSK3, and beta-catenin, by Western blot. **B:** CHO cells were transfected with WT-Mcl-1 and, on the following day, either left untreated or exposed to LiCl (20 mM) or NaCl (20 mM) and assayed after 18 hours for expression of Mcl-1, beta-catenin, and GAPDH (upper panel). In another experiment, CHO cells were either left untreated or exposed to Wortmannin (1 µM) or LiCl (20 mM) for 18 hours, or to LY294002 (20 µM) for 3 hours, and assayed for the expression of beta-catenin, GSK3, and GAPDH (lower panel).
(TIF)

Figure S3 G418 selection of WT-Mcl-1-, but not Mcl-1-T163E-, transfected CHO cells results in the outgrowth of continuous cell lines exhibiting Mcl-1 expression in the endogenous range. **A:** In the upper panel, CHO cells that had been transfected with the indicated constructs and maintained in G418 for >1 month were assayed for Mcl-1 expression by flow cytometry with FITC-conjugated antiMcl-1. The net mean fluorescence index (MFI) for the WT-Mcl-1 and Mcl-1-T163A transfectants (black filled histograms) was 47 and 58, respectively, as compared to values of 130–133 for BL41-3 cells and a previously described clonal transfectant line (WT-Mcl-1-Clone 10 [43]. In the lower panel, CHO cells transfected with Mcl-1-T163E were replated the day after transfection (when ∼80% were viable), and assayed for Mcl-1 expression 24 hours later (black filled histogram). At this time, ∼44% of the cell population exhibited Mcl-1 expression, as estimated by comparison to unstained cells [histograms outlined in light gray which represent unstained Mcl-1-Clone 10 cells, where autofluorescence did not differ for the different lines]. **B:** CHO cells were either left untransfected or transfected with WT-Mcl-1 or Mcl-1-T163E (in the presence of pEGFP). One day later, cells were replated in the absence or presence of G418 (600 micrograms/ml; Day 0) as described [43]. On subsequent days, viable cell number was assayed (trypan blue dye exclusion [53]), where the symbols represent the total viable cell number in each culture (filled symbols represent cultures subjected to G418 selection while open symbols represent parallel cultures not exposed to G418). Viable cells represented a percentage of total (viable plus dead) cells, and this percentage is shown in parentheses. This value is shown at the top of the y-axis for cells examined just prior to G418 addition (day 0 for G418 addition, the day after the start of transfection). The experiment shown is representative of 2 independent experiments. **C:** In the experiment in Panel B, expression of the introduced Mcl-1 gene product or endogenous tubulin was assayed by Western blotting.
(TIF)

Figure S4 TPA-induced Mcl-1 stabilization and increased drug resistance are maintained after 24 hours of exposure of BL41-3 cells. **A:** BL41-3 cells were incubated in the absence or presence of TPA (5 nM) for 0.5 hours prior to the addition of the indicated concentrations of Ara-C. After 24 hours, PARP cleavage and Mcl-1 expression were assayed. **B:** BL41-3 cells were incubated in the absence or presence of TPA (5 nM) for 0.5 hours prior to the addition of the indicated concentrations of vinblastine. After 24 hours, PARP cleavage and Mcl-1 expression were assayed. The blot shown is representative of 2 independent experiments, where PARP cleavage was ≥85% at concentrations of 1–33 micromolar vinblastine but averaged 68% when TPA was also present.
(TIF)

## References

[1] Craig RW (2002) MCL1 provides a window on the role of the BCL2 family in cell proliferation, differentiation and tumorigenesis. Leukemia: 16(4): 444–454 doi:10.1038/sj.leu.2402416. 11960321 10.1038/sj.leu.2402416

[2] Chao JR, Wang JM, Lee SF, Peng HW, Lin YH, et al (1998) Mcl-1 is an immediate-early gene activated by the granulocyte-macrophage colony-stimulating factor (GM-CSF) signaling pathway and is one component of the GM-CSF viability response. Mol Cell Biol: 18(8): 4883–4898. 9671497 10.1128/mcb.18.8.4883 PMC109073

[3] Huang HM, Huang CJ, Yen JJ (2000) Mcl-1 is a common target of stem cell factor and interleukin-5 for apoptosis prevention activity via MEK/MAPK and PI-3K/akt pathways. Blood: 96(5): 1764–1771. 10961875

[4] Derouet M, Thomas L, Cross A, Moots RJ, Edwards SW (2004) Granulocyte macrophage colony-stimulating factor signaling and proteasome inhibition delay neutrophil apoptosis by increasing the stability of mcl-1. J Biol Chem 279(26): 26915–26921. 10.1074/jbc.M313875200. 10.1074/jbc.M313875200 15078892

[5] Nijhawan D, Fang M, Traer E, Zhong Q, Gao W, et al (2003) Elimination of mcl-1 is required for the initiation of apoptosis following ultraviolet irradiation. Genes Dev: 17(12): 1475–1486 doi:10.1101/gad.1093903. 12783855 10.1101/gad.1093903 PMC196078

[6] Cuconati A, Mukherjee C, Perez D, White E (2003) DNA damage response and MCL-1 destruction initiate apoptosis in adenovirus-infected cells. Genes Dev 17(23): 2922–2932. 10.1101/gad.1156903. 10.1101/gad.1156903 PMC289151 14633975

[7] Marriott HM, Bingle CD, Read RC, Braley KE, Kroemer G, et al (2005) Dynamic changes in mcl-1 expression regulate macrophage viability or commitment to apoptosis during bacterial clearance. J Clin Invest 115(2): 359–368. 10.1172/JCI21766. 10.1172/JCI21766 PMC544034 15650769

[8] Konopleva M, Contractor R, Tsao T, Samudio I, Ruvolo PP, et al (2006) Mechanisms of apoptosis sensitivity and resistance to the BH3 mimetic ABT-737 in acute myeloid leukemia. Cancer Cell 10(5): 375–388. 10.1016/j.ccr.2006.10.006. 10.1016/j.ccr.2006.10.006 17097560

[9] van Delft MF, Wei AH, Mason KD, Vandenberg CJ, Chen L, et al (2006) The BH3 mimetic ABT-737 targets selective bcl-2 proteins and efficiently induces apoptosis via bak/bax if mcl-1 is neutralized. Cancer Cell 10(5): 389–399. 10.1016/j.ccr.2006.08.027. 10.1016/j.ccr.2006.08.027 PMC2953559 17097561

[10] Tahir SK, Yang X, Anderson MG, Morgan-Lappe SE, Sarthy AV, et al (2007) Influence of bcl-2 family members on the cellular response of small-cell lung cancer cell lines to ABT-737. Cancer Res 67(3): 1176–1183. 10.1158/0008–5472.CAN-06-2203. 10.1158/0008-5472.CAN-06-2203 17283153

[11] Kaufmann SH, Karp JE, Svingen PA, Krajewski S, Burke PJ, et al (1998) Elevated expression of the apoptotic regulator mcl-1 at the time of leukemic relapse. Blood: 91(3): 991–1000. 9446661

[12] Awan FT, Kay NE, Davis ME, Wu W, Geyer SM, et al (2009) Mcl-1 expression predicts progression-free survival in chronic lymphocytic leukemia patients treated with pentostatin, cyclophosphamide, and rituximab. Blood 113(3): 535–537. 10.1182/blood–2008-08-173450. 10.1182/blood-2008-08-173450 PMC2628361 19008456

[13] Pepper C, Lin TT, Pratt G, Hewamana S, Brennan P, et al (2008) Mcl-1 expression has in vitro and in vivo significance in chronic lymphocytic leukemia and is associated with other poor prognostic markers. Blood 112(9): 3807–3817. 10.1182/blood–2008-05-157131. 10.1182/blood-2008-05-157131 18599795

[14] Sano M, Nakanishi Y, Yagasaki H, Honma T, Oinuma T, et al (2005) Overexpression of anti-apoptotic mcl-1 in testicular germ cell tumours. Histopathology 46(5): 532–539. 10.1111/j.1365-2559.2005.02118.x. 10.1111/j.1365-2559.2005.02118.x 15842635

[15] Maeta Y, Tsujitani S, Matsumoto S, Yamaguchi K, Tatebe S, et al (2004) Expression of mcl-1 and p53 proteins predicts the survival of patients with T3 gastric carcinoma. Gastric Cancer 7(2): 78–84. 10.1007/s10120–004-0272-9. 10.1007/s10120-004-0272-9 15224193

[16] MacCallum DE, Melville J, Frame S, Watt K, Anderson S, et al (2005) Seliciclib (CYC202, R-roscovitine) induces cell death in multiple myeloma cells by inhibition of RNA polymerase II-dependent transcription and down-regulation of mcl-1. Cancer Res 65(12): 5399–5407. 10.1158/0008–5472.CAN-05-0233. 10.1158/0008-5472.CAN-05-0233 15958589

[17] Vrana JA, Cleaveland ES, Eastman A, Craig RW (2006) Inducer-and cell type-specific regulation of antiapoptotic MCL1 in myeloid leukemia and multiple myeloma cells exposed to differentiation-inducing or microtubule-disrupting agents. Apoptosis 11(8): 1275–1288. 10.1007/s10495–006-7787-y. 10.1007/s10495-006-7787-y 16761109

[18] Salerni BL, Bates DJ, Albershardt TC, Lowrey CH, Eastman A (2010) Vinblastine induces acute, cell cycle phase-independent apoptosis in some leukemias and lymphomas and can induce acute apoptosis in others when mcl-1 is suppressed. Mol Cancer Ther 9(4): 791–802. 10.1158/1535–7163.MCT-10-0028. 10.1158/1535-7163.MCT-10-0028 PMC2852489 20371726

[19] Mills JR, Hippo Y, Robert F, Chen SM, Malina A, et al (2008) mTORC1 promotes survival through translational control of mcl-1. Proc Natl Acad Sci U S A 105(31): 10853–10858. 10.1073/pnas.0804821105. 10.1073/pnas.0804821105 PMC2504845 18664580

[20] Ding Q, Huo L, Yang JY, Xia W, Wei Y, et al (2008) Down-regulation of myeloid cell leukemia-1 through inhibiting erk/pin 1 pathway by sorafenib facilitates chemosensitization in breast cancer. Cancer Res 68(15): 6109–6117. 10.1158/0008–5472.CAN-08-0579. 10.1158/0008-5472.CAN-08-0579 PMC2676572 18676833

[21] Kozopas KM, Yang T, Buchan HL, Zhou P, Craig RW (1993) MCL1, a gene expressed in programmed myeloid cell differentiation, has sequence similarity to BCL2. Proc Natl Acad Sci U S A: 90(8): 3516–3520. 7682708 10.1073/pnas.90.8.3516 PMC46331

[22] Yang T, Buchan HL, Townsend KJ, Craig RW (1996) MCL-1, a member of the BLC-2 family, is induced rapidly in response to signals for cell differentiation or death, but not to signals for cell proliferation. J Cell Physiol 166(3): 523–536: 2–R. 10.1002/(SICI)1097-4652(199603)166:3<523::AID-JCP7>3.0.CO;2-R 8600156

[23] Vrana JA, Bieszczad CK, Cleaveland ES, Ma Y, Park JP, et al (2002) An MCL1-overexpressing burkitt lymphoma subline exhibits enhanced survival on exposure to serum deprivation, topoisomerase inhibitors, or staurosporine but remains sensitive to 1-beta-D-arabinofuranosylcytosine. Cancer Res: 62(3): 892–900. 11830549

[24] Domina AM, Smith JH, Craig RW (2000) Myeloid cell leukemia 1 is phosphorylated through two distinct pathways, one associated with extracellular signal-regulated kinase activation and the other with G2/M accumulation or protein phosphatase 1/2A inhibition. J Biol Chem 275(28): 21688–21694.10.1074/jbc.M000915200. 10.1074/jbc.M000915200 10777489

[25] Domina AM, Vrana JA, Gregory MA, Hann SR, Craig RW (2004) MCL1 is phosphorylated in the PEST region and stabilized upon ERK activation in viable cells, and at additional sites with cytotoxic okadaic acid or taxol. Oncogene 23(31): 5301–5315. 10.1038/sj.onc.1207692. 10.1038/sj.onc.1207692 15241487

[26] Yang T, Kozopas KM, Craig RW (1995) The intracellular distribution and pattern of expression of mcl-1 overlap with, but are not identical to, those of bcl-2. J Cell Biol: 128(6): 1173–1184. 7896880 10.1083/jcb.128.6.1173 PMC2120408

[27] Morel C, Carlson SM, White FM, Davis RJ (2009) Mcl-1 integrates the opposing actions of signaling pathways that mediate survival and apoptosis. Mol Cell Biol 29(14): 3845–3852. 10.1128/MCB.00279–09. 10.1128/MCB.00279-09 PMC2704749 19433446

[28] Ding Q, He X, Hsu JM, Xia W, Chen CT, et al (2007) Degradation of mcl-1 by beta-TrCP mediates glycogen synthase kinase 3-induced tumor suppression and chemosensitization. Mol Cell Biol 27(11): 4006–4017. 10.1128/MCB.00620–06. 10.1128/MCB.00620-06 PMC1900029 17387146

[29] Zhao Y, Altman BJ, Coloff JL, Herman CE, Jacobs SR, et al (2007) Glycogen synthase kinase 3alpha and 3beta mediate a glucose-sensitive antiapoptotic signaling pathway to stabilize mcl-1. Mol Cell Biol 27(12): 4328–4339. 10.1128/MCB.00153–07. 10.1128/MCB.00153-07 PMC1900055 17371841

[30] De Biasio A, Vrana JA, Zhou P, Qian L, Bieszczad CK, et al (2007) N-terminal truncation of antiapoptotic MCL1, but not G2/M-induced phosphorylation, is associated with stabilization and abundant expression in tumor cells. J Biol Chem 282(33): 23919–23936. 10.1074/jbc.M700938200. 10.1074/jbc.M700938200 17561513

[31] Maurer U, Charvet C, Wagman AS, Dejardin E, Green DR (2006) Glycogen synthase kinase-3 regulates mitochondrial outer membrane permeabilization and apoptosis by destabilization of MCL-1. Mol Cell 21(6): 749–760. 10.1016/j.molcel.2006.02.009. 10.1016/j.molcel.2006.02.009 16543145

[32] Ding Q, He X, Xia W, Hsu JM, Chen CT, et al (2007) Myeloid cell leukemia-1 inversely correlates with glycogen synthase kinase-3beta activity and associates with poor prognosis in human breast cancer. Cancer Res 67(10): 4564–4571. 10.1158/0008–5472.CAN-06-1788. 10.1158/0008-5472.CAN-06-1788 17495324

[33] Wertz IE, Kusam S, Lam C, Okamoto T, Sandoval W, et al (2011) Sensitivity to antitubulin chemotherapeutics is regulated by MCL1 and FBW7. Nature 471(7336): 110–114. 10.1038/nature09779. 10.1038/nature09779 21368834

[34] Inuzuka H, Shaik S, Onoyama I, Gao D, Tseng A, et al (2011) SCF(FBW7) regulates cellular apoptosis by targeting MCL1 for ubiquitylation and destruction. Nature 471(7336): 104–109. 10.1038/nature09732. 10.1038/nature09732 PMC3076007 21368833

[35] Zhong Q, Gao W, Du F, Wang X (2005) Mule/ARF-BP1, a BH3-only E3 ubiquitin ligase, catalyzes the polyubiquitination of mcl-1 and regulates apoptosis. Cell 121(7): 1085–1095. 10.1016/j.cell.2005.06.009. 10.1016/j.cell.2005.06.009 15989957

[36] Warr MR, Mills JR, Nguyen M, Lemaire-Ewing S, Baardsnes J, et al (2011) Mitochondrion-dependent N-terminal processing of outer membrane mcl-1 protein removes an essential mule/Lasu1 protein-binding site. J Biol Chem 286(28): 25098–25107. 10.1074/jbc.M111.218321. 10.1074/jbc.M111.218321 PMC3137083 21613222

[37] Harley ME, Allan LA, Sanderson HS, Clarke PR (2010) Phosphorylation of mcl-1 by CDK1-cyclin B1 initiates its Cdc20-dependent destruction during mitotic arrest. EMBO J 29(14): 2407–2420. 10.1038/emboj.2010.112. 10.1038/emboj.2010.112 PMC2910263 20526282

[38] Stewart DP, Koss B, Bathina M, Perciavalle RM, Bisanz K, et al (2010) Ubiquitin-independent degradation of antiapoptotic MCL-1. Mol Cell Biol 30(12): 3099–3110. 10.1128/MCB.01266–09. 10.1128/MCB.01266-09 PMC2876674 20385764

[39] Baudot AD, Jeandel PY, Mouska X, Maurer U, Tartare-Deckert S, et al (2009) The tyrosine kinase syk regulates the survival of chronic lymphocytic leukemia B cells through PKCdelta and proteasome-dependent regulation of mcl-1 expression. Oncogene 28(37): 3261–3273. 10.1038/onc.2009.179. 10.1038/onc.2009.179 19581935

[40] Gobessi S, Laurenti L, Longo PG, Carsetti L, Berno V, et al (2009) Inhibition of constitutive and BCR-induced syk activation downregulates mcl-1 and induces apoptosis in chronic lymphocytic leukemia B cells. Leukemia 23(4): 686–697. 10.1038/leu.2008.346. 10.1038/leu.2008.346 19092849

[41] Schwickart M, Huang X, Lill JR, Liu J, Ferrando R, et al (2010) Deubiquitinase USP9X stabilizes MCL1 and promotes tumour cell survival. Nature 463(7277): 103–107. 10.1038/nature08646. 10.1038/nature08646 20023629

[42] Liao M, Zhao J, Wang T, Duan J, Zhang Y, et al (2011) Role of bile salt in regulating mcl-1 phosphorylation and chemoresistance in hepatocellular carcinoma cells. Mol Cancer 10: 44 10.1186/1476–4598-10-44. 10.1186/1476-4598-10-44 PMC3107804 21507240

[43] Reynolds JE, Yang T, Qian L, Jenkinson JD, Zhou P, et al (1994) Mcl-1, a member of the bcl-2 family, delays apoptosis induced by c-myc overexpression in chinese hamster ovary cells. Cancer Res: 54(24): 6348–6352. 7987827

[44] Bissonnette RP, Echeverri F, Mahboubi A, Green DR (1992) Apoptotic cell death induced by c-myc is inhibited by bcl-2. Nature 359(6395): 552–554. 10.1038/359552a0. 10.1038/359552a0 1406975

[45] Becker NA, Kelm RJ Jr, Vrana JA, Getz MJ, Maher LJ 3rd (2000) Altered sensitivity to single-strand-specific reagents associated with the genomic vascular smooth muscle alpha-actin promoter during myofibroblast differentiation. J Biol Chem 275(20): 15384–15391. 10.1074/jbc.M909687199. 10.1074/jbc.M909687199 10748152

[46] Kobayashi S, Lee SH, Meng XW, Mott JL, Bronk SF, et al (2007) Serine 64 phosphorylation enhances the antiapoptotic function of mcl-1. J Biol Chem 282(25): 18407–18417. 10.1074/jbc.M610010200. 10.1074/jbc.M610010200 17463001

[47] Kerr JF, Wyllie AH, Currie AR (1972) Apoptosis: A basic biological phenomenon with wide-ranging implications in tissue kinetics. Br J Cancer: 26(4): 239–257. 4561027 10.1038/bjc.1972.33 PMC2008650

[48] Craig RW, Frankfurt OS, Sakagami H, Takeda K, Bloch A (1984) Macromolecular and cell cycle effects of different classes of agents inducing the maturation of human myeloblastic leukemia (ML-1) cells. Cancer Res: 44(6): 2421–2429. 6586286

[49] Townsend KJ, Trusty JL, Traupman MA, Eastman A, Craig RW (1998) Expression of the antiapoptotic MCL1 gene product is regulated by a mitogen activated protein kinase-mediated pathway triggered through microtubule disruption and protein kinase C. Oncogene 17(10). 1223–1234. 10.1038/sj.onc.1202035. 10.1038/sj.onc.1202035 9771965

[50] Vilimek D, Duronio V (2006) Cytokine-stimulated phosphorylation of GSK-3 is primarily dependent upon PKCs, not PKB. Biochem Cell Biol 84(1): 20–29. 10.1139/o05–154. 10.1139/o05-154 16462886

[51] Ding Q, Xia W, Liu JC, Yang JY, Lee DF, et al (2005) Erk associates with and primes GSK-3beta for its inactivation resulting in upregulation of beta-catenin. Mol Cell 19(2): 159–170. 10.1016/j.molcel.2005.06.009. 10.1016/j.molcel.2005.06.009 16039586

[52] Yewdell JW, Lacsina JR, Rechsteiner MC, Nicchitta CV (2011) Out with the old, in with the new? comparing methods for measuring protein degradation. Cell Biol Int 35(5): 457–462. 10.1042/CBI20110055. 10.1042/CBI20110055 PMC3727619 21476986

[53] Zhou P (2004) Determining protein half-lives. In: Dickson RC MM, editor. Signal Transduction Protocols. Humana Press. 67.

[54] Fujise K, Zhang D, Liu J, Yeh ET (2000) Regulation of apoptosis and cell cycle progression by MCL1. differential role of proliferating cell nuclear antigen. J Biol Chem 275(50): 39458–39465. 10.1074/jbc.M006626200. 10.1074/jbc.M006626200 10978339

[55] Jamil S, Sobouti R, Hojabrpour P, Raj M, Kast J, et al (2005) A proteolytic fragment of mcl-1 exhibits nuclear localization and regulates cell growth by interaction with Cdk1. Biochem J 387(Pt 3): 659–667. 10.1042/BJ20041596. 10.1042/BJ20041596 PMC1134995 15554878

[56] Zhou P, Qian L, Kozopas KM, Craig RW (1997) Mcl-1, a bcl-2 family member, delays the death of hematopoietic cells under a variety of apoptosis-inducing conditions. Blood: 89(2): 630–643. 9002967

[57] Mazumder S, Choudhary GS, Al-Harbi S, Almasan A (2012) Mcl-1 phosphorylation defines ABT-737 resistance that can be overcome by increased NOXA expression in leukemic B cells. Cancer Res 72(12): 3069–3079. 10.1158/0008–5472.CAN-11-4106. 10.1158/0008-5472.CAN-11-4106 PMC3377792 22525702

[58] Jin L, Hu WL, Jiang CC, Wang JX, Han CC, et al (2011) MicroRNA-149*, a p53-responsive microRNA, functions as an oncogenic regulator in human melanoma. Proc Natl Acad Sci U S A 108(38): 15840–15845. 10.1073/pnas.1019312108. 10.1073/pnas.1019312108 PMC3179083 21896753

