# Supplementary figures and images for: Thr 163 Phosphorylation Causes Mcl-1 Stabilization when Degradation Is Independent of the Adjacent GSK3-Targeted Phosphodegron, Promoting Drug Resistance in Cancer
Source: PLoS One. 2012 Oct 9;7(10):e47060. doi: 10.1371/journal.pone.0047060 (PMC3467206; doi:10.1371/journal.pone.0047060)

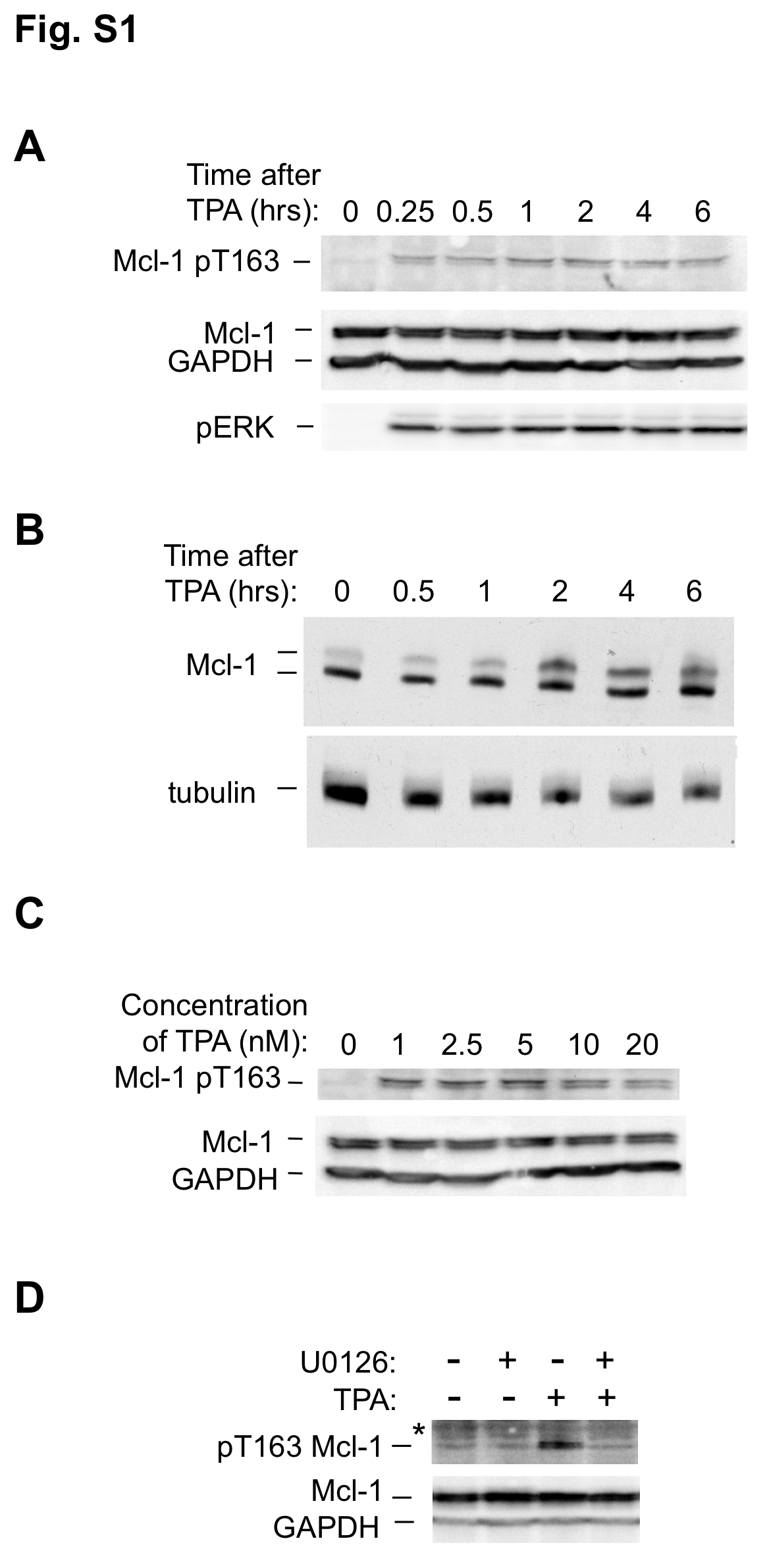

Supplement: Figure S1 — Exposure of BL41-3 cells to TPA to activate ERK stimulates Thr 163 phosphorylation in a fashion that is U0126-inhibitable but does not involve a substantial increase in Mcl-1 expression. A: BL41-3 cells were treated with 5 nM TPA and assayed for expression of Thr 163 phosphorylated Mcl-1 (Mcl-1 pT163), total Mcl-1, GAPDH, and phospho-ERK at the indicated times by Western blotting (ChemiDoc). B: BL41-3 cells were treated with 5 nM TPA and assayed for Mcl-1 expression using a large format gel that separates the 42/40 kd Mcl-1 doublet bands. C: BL41-3 cells were treated with the indicated concentrations of TPA and assayed for expression of Thr 163 phosphorylated Mcl-1, total Mcl-1, and GAPDH after 1 hour as in Panel A. D: BL41-3 cells were incubated in the absence or presence of U0126 (25 mM) for 30 minutes, and TPA (5 nM) was then added as indicated for an additional 3 hours. Expression of Thr 163 phosphorylated Mcl-1, total Mcl-1 and GAPDH was assessed as in Panel A. The asterisk indicates a non-specific band. (TIF) [file pone.0047060.s001.tif]

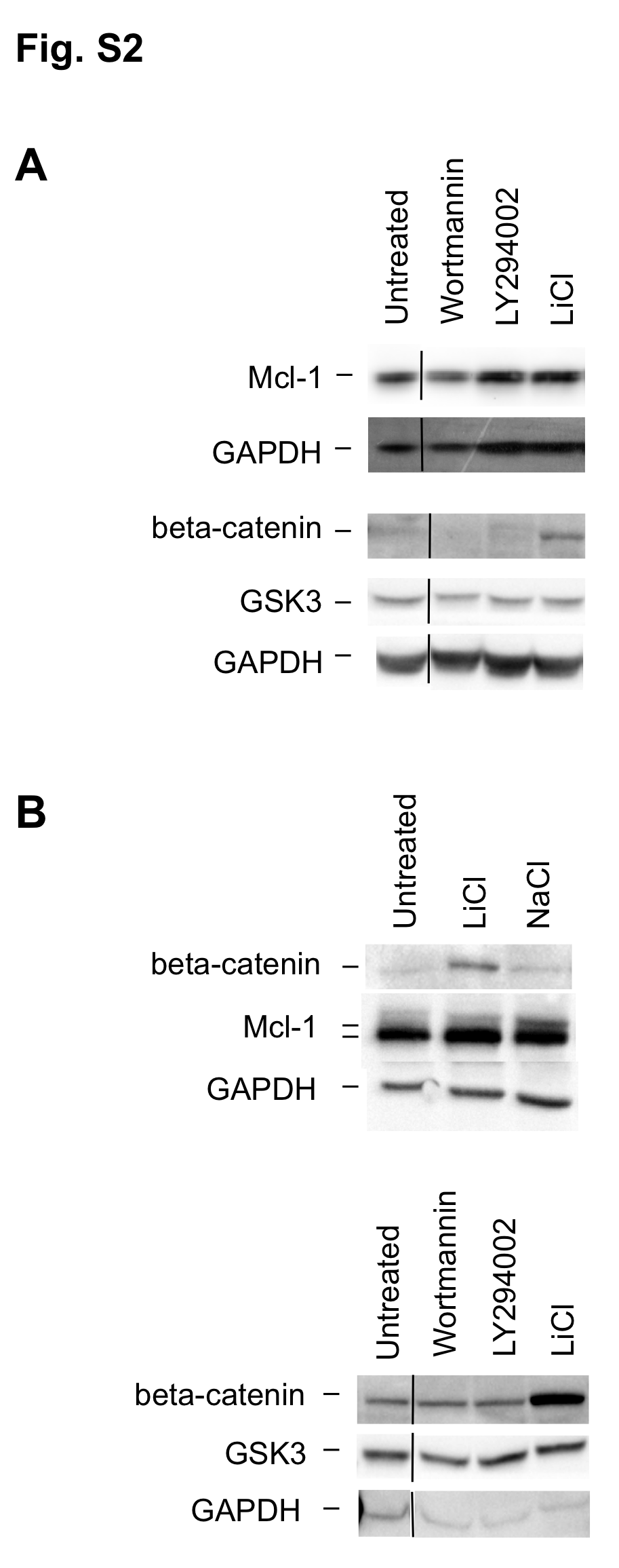

Supplement: Figure S2 — LiCl does not affect Mcl-1 expression in BL41-3 cells or WT-Mcl-1-transfected CHO cells. A: BL41-3 cells were either left untreated or exposed to Wortmannin (1 µM) or LiCl (20 mM) for 18 hours, or to LY294002 (20 µM) for 3 hours, and then assayed for the expression of Mcl-1, GAPDH, GSK3, and beta-catenin, by Western blot. B: CHO cells were transfected with WT-Mcl-1 and, on the following day, either left untreated or exposed to LiCl (20 mM) or NaCl (20 mM) and assayed after 18 hours for expression of Mcl-1, beta-catenin, and GAPDH (upper panel). In another experiment, CHO cells were either left untreated or exposed to Wortmannin (1 µM) or LiCl (20 mM) for 18 hours, or to LY294002 (20 µM) for 3 hours, and assayed for the expression of beta-catenin, GSK3, and GAPDH (lower panel). (TIF) [file pone.0047060.s002.tif]

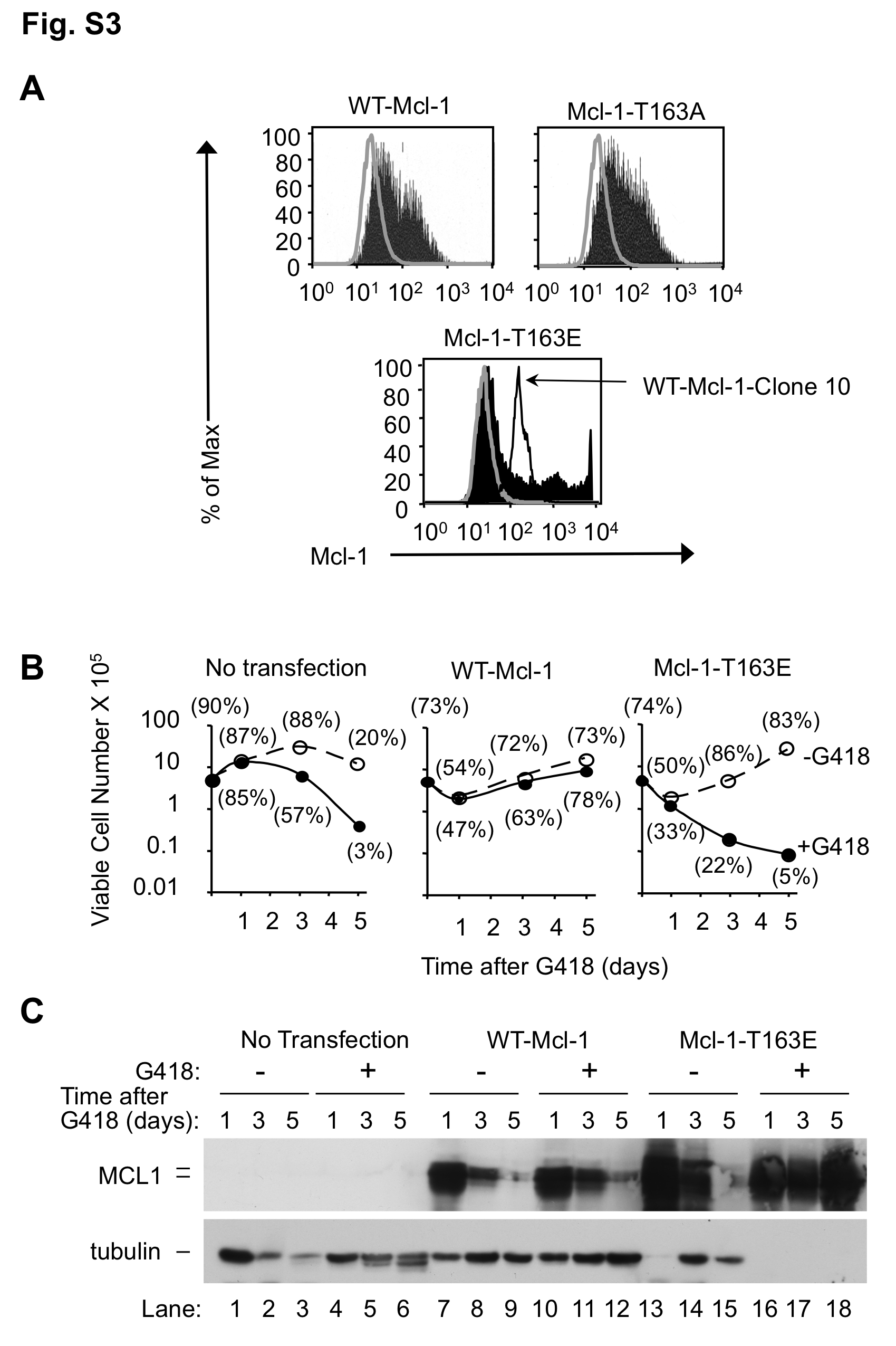

Supplement: Figure S3 — G418 selection of WT-Mcl-1-, but not Mcl-1-T163E-, transfected CHO cells results in the outgrowth of continuous cell lines exhibiting Mcl-1 expression in the endogenous range. A: In the upper panel, CHO cells that had been transfected with the indicated constructs and maintained in G418 for >1 month were assayed for Mcl-1 expression by flow cytometry with FITC-conjugated antiMcl-1. The net mean fluorescence index (MFI) for the WT-Mcl-1 and Mcl-1-T163A transfectants (black filled histograms) was 47 and 58, respectively, as compared to values of 130–133 for BL41-3 cells and a previously described clonal transfectant line (WT-Mcl-1-Clone 10 [43]. In the lower panel, CHO cells transfected with Mcl-1-T163E were replated the day after transfection (when ∼80% were viable), and assayed for Mcl-1 expression 24 hours later (black filled histogram). At this time, ∼44% of the cell population exhibited Mcl-1 expression, as estimated by comparison to unstained cells [histograms outlined in light gray which represent unstained Mcl-1-Clone 10 cells, where autofluorescence did not differ for the different lines]. B: CHO cells were either left untransfected or transfected with WT-Mcl-1 or Mcl-1-T163E (in the presence of pEGFP). One day later, cells were replated in the absence or presence of G418 (600 micrograms/ml; Day 0) as described [43]. On subsequent days, viable cell number was assayed (trypan blue dye exclusion [53]), where the symbols represent the total viable cell number in each culture (filled symbols represent cultures subjected to G418 selection while open symbols represent parallel cultures not exposed to G418). Viable cells represented a percentage of total (viable plus dead) cells, and this percentage is shown in parentheses. This value is shown at the top of the y-axis for cells examined just prior to G418 addition (day 0 for G418 addition, the day after the start of transfection). The experiment shown is representative of 2 independent experiments. C: In the experim [file pone.0047060.s003.tif]

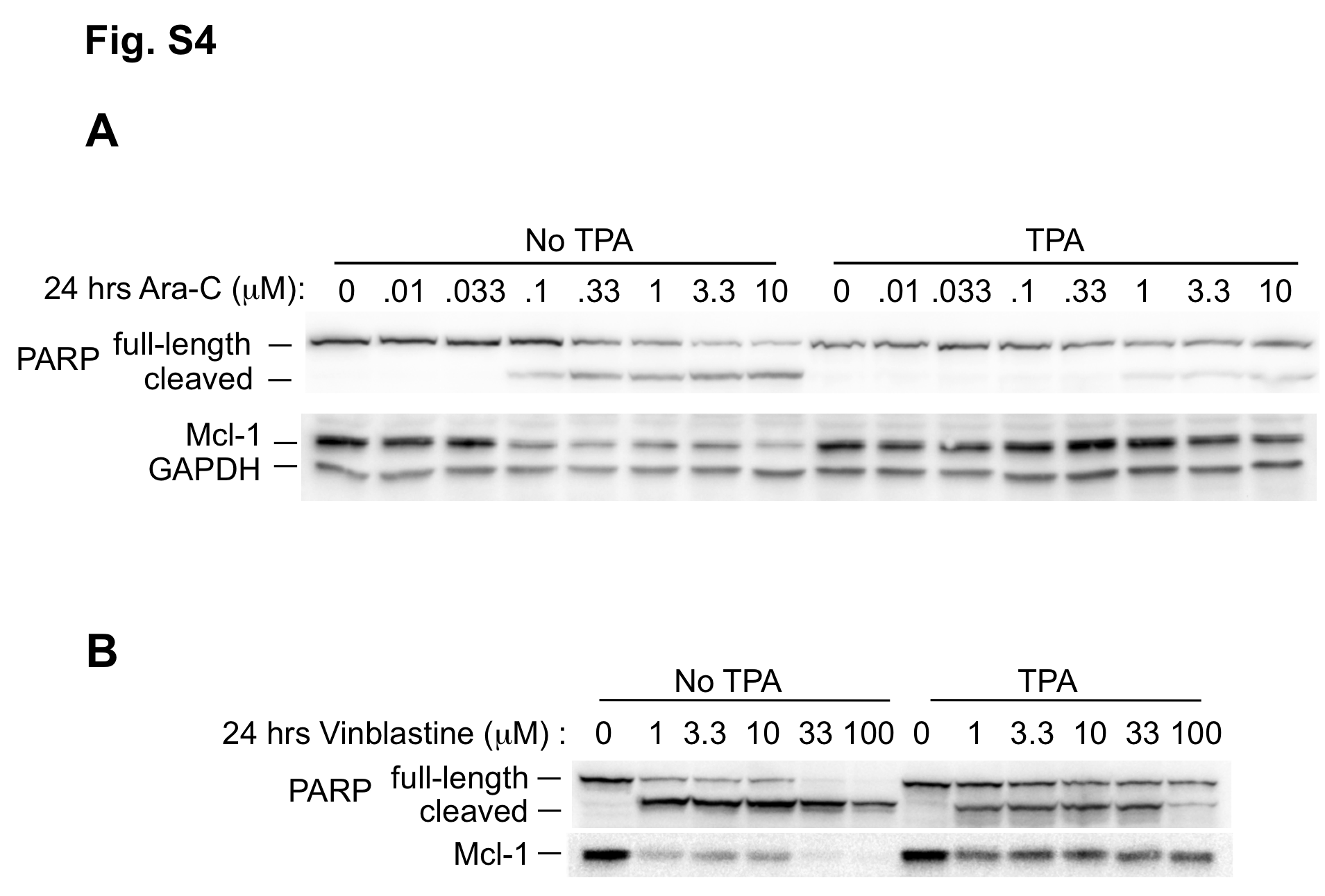

Supplement: Figure S4 — TPA-induced Mcl-1 stabilization and increased drug resistance are maintained after 24 hours of exposure of BL41-3 cells. A: BL41-3 cells were incubated in the absence or presence of TPA (5 nM) for 0.5 hours prior to the addition of the indicated concentrations of Ara-C. After 24 hours, PARP cleavage and Mcl-1 expression were assayed. B: BL41-3 cells were incubated in the absence or presence of TPA (5 nM) for 0.5 hours prior to the addition of the indicated concentrations of vinblastine. After 24 hours, PARP cleavage and Mcl-1 expression were assayed. The blot shown is representative of 2 independent experiments, where PARP cleavage was ≥85% at concentrations of 1–33 micromolar vinblastine but averaged 68% when TPA was also present. (TIF) [file pone.0047060.s004.tif]
